# Different milk diets have substantial effects on the jejunal mucosal immune system of pre-weaning calves, as demonstrated by whole transcriptome sequencing

**DOI:** 10.1038/s41598-018-19954-2

**Published:** 2018-01-26

**Authors:** H. M. Hammon, D. Frieten, C. Gerbert, C. Koch, G. Dusel, R. Weikard, C. Kühn

**Affiliations:** 1 Leibniz Institute for Farm Animal Biology (FBN), Dummerstorf, Germany; 2 University of Applied Sciences, Bingen, Germany; 3 Educational and Research Centre for Animal Husbandry, Hofgut Neumühle, Münchweiler, Germany; 4 University Rostock, Faculty of Agricultural and Environmental Sciences, Rostock, Germany

## Abstract

There is increasing evidence that nutrition during early mammalian life has a strong influence on health and performance in later life. However, there are conflicting data concerning the appropriate milk diet. This discrepancy particularly applies to ruminants, a group of mammals that switch from monogastric status to rumination during weaning. Little is known regarding how the whole genome expression pattern in the juvenile ruminant gut is affected by alternative milk diets. Thus, we performed a next-generation-sequencing-based holistic whole transcriptome analysis of the jejunum in male pre-weaned German Holstein calves fed diets with restricted or unlimited access to milk during the first 8 weeks of life. Both groups were provided hay and concentrate *ad libitum*. The analysis of jejunal mucosa samples collected 80 days after birth and four weeks after the end of the feeding regimes revealed 275 differentially expressed loci. While the differentially expressed loci comprised 67 genes encoding proteins relevant to metabolism or metabolic adaptation, the most distinct difference between the two groups was the consistently lower activation of the immune system in calves that experienced restricted milk access compared to calves fed milk *ad libitum*. In conclusion, different early life milk diets had significant prolonged effects on the intestinal immune system.

## Introduction

During the postnatal and pre-weaning periods, mammals must adapt to extrauterine life and change their nutrient metabolism. These changes encompass nutrient digestion and absorption, adaptation to extrauterine energy metabolism, including regulation of body temperature, and the development of immune defence mechanisms^1–3^. In this context, the postnatal development and maturation of the gastrointestinal tract are of prominent importance because of its role in nutrient uptake and as the first line of immune defence^4^. In cattle, further significant changes in gastrointestinal function occur during the pre-weaning period^5,6^. Different feeding strategies affect the gastrointestinal development of pre-ruminant calves and can subsequently have long-lasting consequences on calf performance^7–10^.

Adequate intake of colostrum and milk seems to be of essential importance for the immediate maturation of the intestine, because nutritive and non-nutritive factors in colostrum and milk support the development and functioning of the gut in neonatal calves^11–13^. Intensive milk feeding programmes result in elevated dry matter and energy intake and body growth^14,15^ and accelerate organ, e.g., mammary gland, development^16,17^ during the pre-weaning period. Metabolic and endocrine changes in blood plasma are supportive of apparent increases in anabolic metabolism during intensive milk feeding programmes^18–20^. In addition, there is evidence of improved health and immune function in pre-ruminant calves when milk or milk replacer is fed at a high level (calves fed more than 600 g of powder per day with a protein content higher than 25%)^21–23^. However, less is known about the effects of feeding unlimited amounts of milk or milk replacer to pre-ruminant calves for 5–8 weeks after birth on the subsequent development, maturation and function of the small intestine.

Novel holistic analyses, including whole transcriptome sequencing, have established new perspectives that have revealed previously unknown functions and regulatory responses in target tissues^24^. Especially for non-model organisms with incompletely annotated genomes and with many genes of yet unknown functions, RNA sequencing has provided new insights, e.g., into the immune responses to vaccination and infection^25^. Immune response to non-self antigens is highly species-specific^24^. Ruminants, in particular, display a number of specific physiological and immunological features that differ from those of model species, humans and other livestock. For example, ruminants very effectively convert butyrate into β-hydroxybutyrate after absorption of the compound by ruminal epithelial cells, and cattle show a very high proportion of regulatory T cells that are classified as γ/δ cells compared to other species^26^.

For our analyses of the effects of different neonatal diets, we specifically selected the end of the pre-weaning period because it represents the second major adjustment period of the neonatal gut after birth: the change from a fluid (milk) diet to a solid (concentrate and forage) diet. This period is often associated with a high prevalence of health problems^5,8^, suggesting impairment of the calves’ immune system. Furthermore, at the end of the pre-weaning period, the degree of maturation of the intestine and its capacity for appropriate digestion of a solid diet are of prime importance. Thus, the objective of our study was to evaluate the consequences of two different postnatal feeding regimes on the metabolic, absorptive and immunological functions of intestinal tissue in calves within the critical time period of pre-weaning. The use of whole transcriptome sequencing (RNAseq) made it possible for us to conduct hypothesis-free analyses of gene expression levels in jejunal epithelium to elucidate differences in the responses of young calves to two different, controversially discussed feeding regimens.

## Results

### Feed intake, body and intestinal growth, butyrate concentration

During the entire experiment (for the experimental setup, see Supplementary Fig. S1), milk intake and growth, as well as other parameters (e.g., metabolites; for further details see^27^), were analysed. First colostrum intake (2.5 L) was the same for both groups. During the first six meals, calves in the *ad libitum* group (AL) consumed significantly more colostrum and transition milk than calves with restricted milk and milk replacer access (RES). Similarly, the milk replacer intake in all weeks except the last week prior to sampling was significantly greater in AL animals than in RES animals (see Supplementary Fig. S2). Concentrate intake increased in both groups with time and was greater (*P* < 0.01) from week 7 onward in RES calves than in AL calves (see Supplementary Fig. S2). Total dry matter intake (sum of milk, milk replacer and concentrate intake) for the whole experimental period did not differ between AL and RES calves. However, total dry matter intake was greater in the AL calves than in the RES calves during the first weeks after birth but was lower during the last two weeks prior to the end of the experiment (see Supplementary Fig. S2). Birth weight was the same in the two groups, but body weight increased much more rapidly in AL calves than in RES calves (see Supplementary Fig. S2), and AL calves were heavier (*P* < 0.01) than RES calves from week 4 through week 10 (see Supplementary Fig. S2). The feed conversion ratio (kg gain/kg dry matter intake) was 0.65 ± 0.04 from week 1 to 8 and 0.62 ± 0.07 from week 9 to week 11; it did not differ between the two groups. At slaughter, the small intestine tended to be longer (*P* < 0.1) in AL calves (27.1 ± 1.0 m) than in RES calves (24.2 ± 1.0 m). From week 5 onward, the RES calves had significantly elevated blood plasma β-hydroxybutyrate concentrations compared to AL calves^27^. However, at day 77, blood plasma β-hydroxybutyrate was significantly higher in AL calves (0.24 ± 0.02 mmol/L) than in RES calves (0.17 ± 0.02 mmol/L (LSM ± SE); P < 0.001). Plasma butyrate was below the detection limit in all calves. Rumen butyrate did not differ significantly between the two groups at slaughter (19.1 ± 3.8 mmol/L for AL and 16.5 ± 3.8 mmol/L for RES (LSM ± SE)).

### Statistics of whole transcriptome sequencing

Jejunal epithelium samples from all calves were subjected to paired-end next-generation sequencing of Illumina TruSeq Stranded RNA libraries. After quality control, adapter trimming and quality filtering, a total of 6.8 billion reads (43,051,555 to 70,155,207 fragments per sample; see Supplementary Fig. S3) were submitted for subsequent analyses. After guided alignment to the UMD 3.1 *Bos taurus* genome, 88.6% of the reads could be mapped to the bovine reference assembly. Initial data analysis using multidimensional scaling plots of normalized read counts per gene and sample indicated that the expression profile of the sample from one calf of the AL group (R_6) differed substantially from the others (see Supplementary Fig. S4). Further specific scrutiny of the read count data for tissue-specific genes from this sample indicated that this sample obviously had not been taken from the jejunal mucosa. Retrospective analysis of the sampling protocols confirmed this hypothesis and indicated that the sample comprised the whole jejunal wall including the muscular layer. Consequently, this sample was removed from further analyses. Annotation-guided transcript assembly revealed 14,088 loci with an average expression FPKM (fragments per kilo base transcript per million reads) value >1 in at least one of the two groups; 2,269 of those were not annotated in the *Bos taurus* Ensembl genome annotation v. 83.

### Most highly expressed loci

The most highly expressed genes with respect to mean FPKM in the two groups are indicated in Table 1. In the AL calves, the four loci with the highest gene expression were *FABP1*, *APOA1*, *FABP2*, and *APOC3* (see Supplementary Table S1). Interestingly, loci encoding MHC molecules (*B2M*, *BoLa*) and loci encoding molecules with protective functions against exogenous agents (*MUC13*, *S100A*) were also very highly expressed. A very similar pattern with only minor re-ranking of genes was observed for the RES calves (see Supplementary Table S1). Interestingly, *ENSBTAG00000022715*, which is predicted to be homologous to the human *DMBT1* gene, is very highly expressed in calf jejunum (it is within the top 10 genes for AL and above the upper threshold for quantification via Cuffdiff2 for RES). The function of this locus in cattle was previously unknown. The human *DMBT1* gene was also found to be very highly expressed in jejunal mucosa and there is evidence that the *DMBT1* gene may play a role in the mucosal defence system, in cellular immune defence and in epithelial differentiation^28^.

**Table 1:** List of the 10 most highly expressed annotated genes determined from RNAseq data in jejunal mucosa of pre-weaning calves according to the mean FPKM value across all samples.

Gene	Gene name	FPKM
FABP1	Fatty acid-binding protein 1	54842.4
FABP2	Fatty acid-binding protein 2	32379.1
APOA1	Apolipoprotein A I	28801.5
APOC3	Apolipoprotein C III,	21357.7
S100G	S100 calcium binding protein G	16413.9
B2M	Beta microglobulin	13963.3
RBP2	Retinol binding protein 2, cellular	9684.8
APOA4	Apolipoprotein A-IV	8781.5
BOLA	Bovine MHC	5666.9
MUC13	Mucin 13, cell surface associated	4016.4

FPKM: fragments per kilo base per million reads.

### Overview of differentially expressed loci

The list of genes that were differentially expressed in the two groups of calves (Table 2 and Supplementary Table S2) comprised a total of 275 loci. Of those, 65 initially did not have an annotation in Ensembl 83. Screening of the National Center for Biotechnology Information (NCBI) *Bos taurus* annotation (https://www.ncbi.nlm.nih.gov/projects/mapview/map_search.cgi?taxid=9913&build=104.0) for those 65 loci enabled assignment of a predicted gene identifier for 10 of the loci. Thus, 55 loci for which there was no prior information in publicly available databases displayed differential expression between groups, providing the first experimental support for their expression and some indication of their potential function.

**Table 2:** List of the twenty most significantly expressed genes (sorted first by q value and subsequently by log2 ratio of expression AL vs. RES) in jejunal mucosa of pre-weaning calves fed *ad libitum* (AL) or restricted (RES) milk (replacer) diets.

Symbol	Entrez Gene Name	q-value	Exp Log Ratio	Location	Type(s)
B4GALNT2	beta-1,4-N-acetyl-galactosaminyltransferase 2	0.009	8.887	Cytoplasm	enzyme
CYP4B1	cytochrome P450 family 4 subfamily B member 1	0.009	−4.265	Cytoplasm	enzyme
MT1A	metallothionein 1A	0.009	−3.760	Cytoplasm	other
GSTO1	glutathione S-transferase omega 1	0.009	−3.680	Cytoplasm	enzyme
GAL3ST2	galactose-3-O-sulfotransferase 2	0.009	3.347	Cytoplasm	enzyme
OLFM4	olfactomedin 4	0.009	3.264	Extracellular Space	other
CRYAB	crystallin alpha B	0.009	−3.190	Nucleus	other
ANXA10	annexin A10	0.009	3.168	Cytoplasm	other
TMEFF1	transmembrane protein with EGF like and two follistatin-like domains 1	0.009	3.027	Plasma Membrane	other
IDO2	indoleamine 2,3-dioxygenase 2	0.009	−2.972	Cytoplasm	enzyme
CPA1	carboxypeptidase A1	0.009	−2.858	Extracellular Space	peptidase
MACC1	metastasis associated in colon cancer 1	0.009	2.700	Nucleus	other
CA9	carbonic anhydrase 9	0.009	−2.632	Nucleus	enzyme
CPO	carboxypeptidase O	0.009	−2.606	Plasma Membrane	enzyme
REM1	RAS (RAD and GEM)-like GTP-binding 1	0.009	−2.604	Other	enzyme
PMP22	peripheral myelin protein 22	0.009	−2.453	Plasma Membrane	other
AQP7	aquaporin 7	0.009	−2.418	Plasma Membrane	transporter
RCAN1	regulator of calcineurin 1	0.009	−2.222	Nucleus	transcription regulator
ADCY6	adenylate cyclase 6	0.009	2.211	Plasma Membrane	enzyme
KBTBD7	kelch repeat and BTB domain containing 7	0.009	−2.188	Cytoplasm	other

The list of differentially expressed known genes that was uploaded for subsequent pathway and network analyses comprises 10 loci classified as cytokines (mostly C-C or C-X-C motif chemokine ligands), 40 loci encoding enzymes, 10 loci encoding ion channels, seven kinases, 11 peptidases, four phosphatases, 16 transcription factors, seven transmembrane receptors, and 18 transporters. The most highly differentially expressed genes include a substantial number of loci encoding proteins with enzymatic activity (*B4GALNT2*, encoding beta-1,4-N-acetyl-galactosaminyltransferase 2, *GSTO1*, encoding glutathione S-transferase omega 1, *GAL3ST2*, encoding galactose-3-O-sulfotransferase 2, and *CPA1*, encoding carboxypeptidase A1; Table 2). Although a number of genes that encode transporter proteins were also differentially expressed, their respective fold changes were not within the highest order of magnitude.

### Enrichment analyses of GO terms, KEGG pathways and Ingenuity canonical pathways with differentially expressed genes

The list of differentially expressed genes was subsequently subjected to enrichment analyses. Within the pathways listed in the KEGG database (Kyoto Encyclopedia of Genes and Genomes, http://www.genome.jp/kegg/), complement and coagulation cascades, toll-like receptor signalling pathways, and prion diseases were all overrepresented in our list of differentially expressed genes (Table 3). An enrichment of factors involved in the immune response was also detected by enrichment analysis based on GO terms: the list of significantly enriched GO terms almost exclusively contains biochemical processes or molecular functions involved in immune or inflammatory responses, including, e.g., immune response and chemokine activity (Table 3).

**Table 3:** Enriched KEGG pathways and GO terms associated with differentially expressed genes in jejuna of calves fed *ad libitum* vs. restricted milk (replacer) diets.

Category	Term	Fold Enrichment	q_Benjamini-Hochberg_
KEGG pathway	hsa04610:Complement and coagulation cascades	8.4	0.003
KEGG pathway	hsa04620:Toll-like receptor signalling pathway	5.8	0.017
KEGG pathway	hsa05020:Prion diseases	10.4	0.035
GOTERM_BP_FAT	GO:0006954~inflammatory response	5.96	6.39E-07
GOTERM_BP_FAT	GO:0009611~response to wounding	4.5	1.21E-06
GOTERM_BP_FAT	GO:0006952~defence response	4.1	1.58E-06
GOTERM_BP_FAT	GO:0002526~acute inflammatory response	10.8	2.12E-05
GOTERM_CC_FAT	GO:0005576~extracellular region	2.0	3.94E-04
GOTERM_CC_FAT	GO:0044421~extracellular region part	2.6	6.97E-04
GOTERM_CC_FAT	GO:0005615~extracellular space	3.1	7.82E-04
GOTERM_BP_FAT	GO:0010033~response to organic substance	3.1	0.001
GOTERM_MF_FAT	GO:0042379~chemokine receptor binding	13.2	0.002
GOTERM_MF_FAT	GO:0008009~chemokine activity	14.0	0.003
GOTERM_BP_FAT	GO:0006955~immune response	2.8	0.019
GOTERM_CC_FAT	GO:0044459~plasma membrane part	1.78	0.020
GOTERM_BP_FAT	GO:0048545~response to steroid hormone stimulus	5.0	0.024
GOTERM_BP_FAT	GO:0010039~response to iron ion	32.0	0.026
GOTERM_BP_FAT	GO:0006958~complement activation, classical pathway	16.5	0.027
GOTERM_BP_FAT	GO:0048584~positive regulation of response to stimulus	4.5	0.027
GOTERM_BP_FAT	GO:0002455~humoural immune response mediated by circulating immunoglobulin	15.5	0.029
GOTERM_BP_FAT	GO:0034097~response to cytokine stimulus	8.5	0.030

Further evidence that the immune system is primarily affected by the different neonatal diets was obtained from Ingenuity Pathway Analysis (IPA). A total of 87 canonical pathways showed significant enrichment for the genes that were differentially expressed between AL and RES. Similar to the KEGG and GO term enrichment analyses, the list of significantly enriched canonical pathways exhibited a large preponderance of pathways associated with the immune response (Table 4 and Supplementary Table S3). Despite the fact that the two experimental groups differed with respect to the diets the animals had received for eight weeks (until 24 days before sampling), none of the top 20 enriched canonical pathways was directly related to energy metabolism or digestion-associated pathways. This might be because the expression of genes associated with pathways related to energy metabolism may predominantly depend on the nutrient and therefore energy intake at or immediately prior to sampling; these parameters differed only slightly in the two groups at the end of the study (Supplementary Figure S2). However, we cannot rule out the possibility that the different diets resulted in prolonged effects on the stem cells in the intestinal crypts. As a consequence, the result seen at sampling might reflect the sum of effects from the time period immediately prior to sampling and prolonged carry-over effects from the time period during which the animals received different diets.

**Table 4:** Twenty most significantly enriched canonical pathways associated with differentially expressed genes in the jejuna of calves fed *ad libitum* vs. restricted milk (replacer) diets as determined by Ingenuity Pathway Analysis.

Ingenuity Canonical Pathway	−log(p-value)	Ratio
Agranulocyte Adhesion and Diapedesis	1.09E01	8.47E-02
Granulocyte Adhesion and Diapedesis	9.17E00	7.91E-02
Pathogenesis of Multiple Sclerosis	8.19E00	5.56E-01
IL-17A Signalling in Gastric Cells	7.15E00	2.4E-01
Complement System	4.74E00	1.35E-01
CCR5 Signalling in Macrophages	4.46E00	8.7E-02
Bupropion Degradation	4.18E00	1.6E-01
VDR/RXR Activation	4.16E00	7.69E-02
Acetone Degradation I (to Methylglyoxal)	4.04E00	1.48E-01
IL-17A Signalling in Fibroblasts	3.59E00	1.14E-01
Oestrogen Biosynthesis	3.41E00	1.03E-01
Chemokine Signalling	3.37E00	7.04E-02
Role of MAPK Signalling in the Pathogenesis of Influenza	3.34E00	6.94E-02
Differential Regulation of Cytokine Production in Macrophages and T Helper Cells by IL-17A and IL-17F	3.29E00	1.67E-01
Toll-like Receptor Signalling	3.29E00	6.76E-02
Role of Hypercytokinemia/hyperchemokinemia in the Pathogenesis of Influenza	3.24E00	9.3E-02
Tight Junction Signalling	3.12E00	4.19E-02
Acute Phase Response Signalling	3.09E00	4.14E-02
Differential Regulation of Cytokine Production in Intestinal Epithelial Cells by IL-17A and IL-17F	2.97E00	1.3E-01
Sertoli Cell-Sertoli Cell Junction Signalling	2.96E00	3.93E-02

### Identification of potential upstream regulators of differentially expressed loci

**Figure 1:**
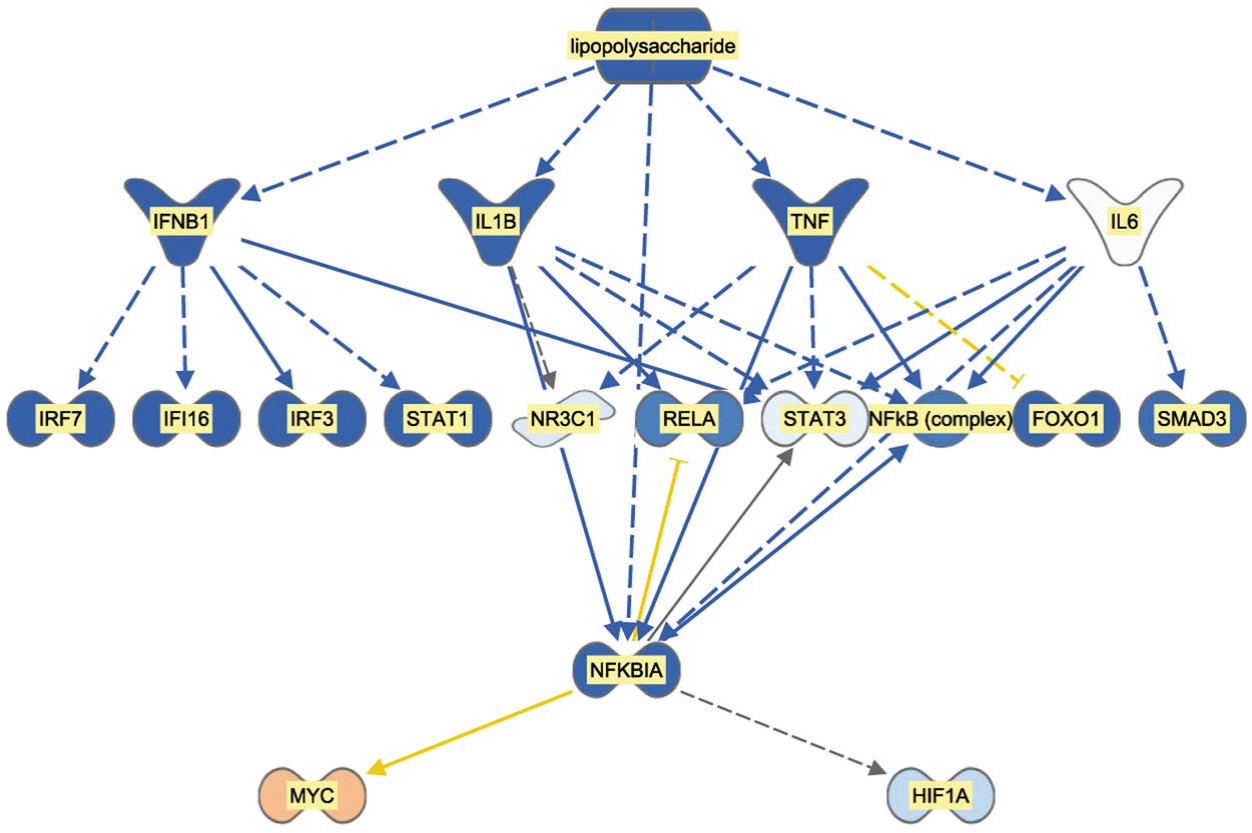
Mechanistic network of upstream regulators in the jejunal epithelium of calves on a restricted milk (replacer) diet compared to calves fed an *ad libitum* diet. Blue symbols indicate predicted inactivation status of the molecule, beige symbols indicate predicted activation, blue lines indicate predicted inactivation, brown lines indicate predicted activation, yellow lines indicate effects that are inconsistent with predictions, and grey lines indicate that no effect direction is predicted.

The IPA analysis of potential inhibited or activated upstream transcriptional regulators comprises any type of molecule (transcription factor, microRNA, compound, drug, etc.) that can modulate biological activities occurring in the studied tissue (https://www.qiagenbioinformatics.com/products/ingenuity-pathway-analysis/). A molecule is classified as an affected upstream regulator if there is a significant enrichment of differentially expressed downstream genes in the data set. The IPA analysis then assigns an activation score to each upstream regulator. In our data set, many molecules that were classified as upstream regulators and were relevant to the immune response displayed very coordinated negative activation z-scores. This indicates that there was lower activation of these upstream regulators in the calves that received the restricted milk replacer diet than in the calves that received the *ad libitum* milk replacer diet (Table 5; also see Supplementary Table S4). For example, the results of the upstream regulator analysis supported the concept that the lipopolysaccharide (LPS) response, as well as the subsequent downstream regulators of LPS signalling, were inactivated or reduced in the calves that received a restricted diet (Fig. 1). Whereas immune response-associated upstream regulators were predominantly indicated as inactivated, the list of upstream regulators with positive activation score comprises almost exclusively microRNAs and single inactivators or suppressors of cytokine signalling and immune response activation. None of the most strongly affected potential upstream regulators (Table 5) was directly associated with the presence of specific micro- or macronutrients in the diet.

**Table 5:** Upstream regulators with the most strongly predicted inhibition determined by Ingenuity Pathway Analysis from the list of differentially expressed genes in the jejuna of calves fed *ad libitum* vs. restricted milk replacer diets.

Upstream Regulator	Molecule type	Predicted activation state	Activation z-score
poly rI:rC-RNA	biologic drug	inhibited	−3.793
ERBB2	kinase	inhibited	−3.238
CXCL12	cytokine	inhibited	−3.098
TNF	cytokine	inhibited	−3.041
IFNG	cytokine	inhibited	−2.996
TLR7	transmembrane receptor	inhibited	−2.945
IFNA2	cytokine	inhibited	−2.936
IFNB1	cytokine	inhibited	−2.925
lipopolysaccharide	chemical drug	inhibited	−2.922
ionomycin	chemical reagent	inhibited	−2.789
TLR3	transmembrane receptor	inhibited	−2.781
FOXO3	transcription regulator	inhibited	−2.777
TCR	complex	inhibited	−2.692
STAT4	transcription regulator	inhibited	−2.621
MAPK8	kinase	inhibited	−2.621
IRF5	transcription regulator	inhibited	−2.596
PPARA	ligand-dependent nuclear receptor	inhibited	−2.59
TLR9	transmembrane receptor	inhibited	−2.589
Growth hormone	hormone	inhibited	−2.578
lactacystin	chemical - protease inhibitor	inhibited	−2.562

## Discussion

In response to the different feeding regimes that were used essentially up to four weeks prior to sampling, the two groups of calves differed in two major features, as revealed by analysis of the whole transcriptome data in the jenunal mucosa. The coordinated differences in immune response were the dominating result of pathway analyses in our data set. The second distinct feature resulting from the two feeding regimes was a different metabolic response in the two groups, although these diet-associated responses were not specifically highlighted in the enrichment analyses.

There is a high consensus in the data across all functional enrichment/pathway analyses (KEGG, GO term, Ingenuity canonical pathways, Ingenuity upstream regulators) indicating that, predominantly, genes acting in pathways related to immune defence were differently modulated in AL and RES calves. This is demonstrated by the very significant enrichment of processes or pathways involved in the immune response. In contrast, only single genes encoding proteins involved in metabolism displayed substantial fold changes in expression levels (e.g., *TRPV6*, the gene encoding transient receptor potential cation channel subfamily V member 6, which mediates Ca^2+^ uptake in the intestine). These effects were observed even after the dietary regimens of the two groups of calves had been fully equalized for two weeks. Because they obviously persisted at the time of sampling, the effects observed at the transcriptional level may be prolonged effects of the different feeding regimes. Alternatively, the observed changes in expression at the transcriptional level of genes involved in metabolism might be due to alterations in the physiological growth status of the animals rather than to a direct influence of the diet. In our experiment, the presence of increased cell proliferation signals is indicated by the higher expression level of *MYB* and the greater activation of the upstream regulator *MYC* in the RES calves (see Fig. 1). However, the hypothesis of compensatory growth in RES calves at the end of the feeding experiment must be carefully evaluated because the RES diet was not restricted in terms of total energy intake but only in terms of energy intake via milk. In addition, the RES diet is currently standard for most dairy calves world-wide; consequently, calves fed this diet were used as the control group to which the AL calves were compared in our study.

**Figure 2:**
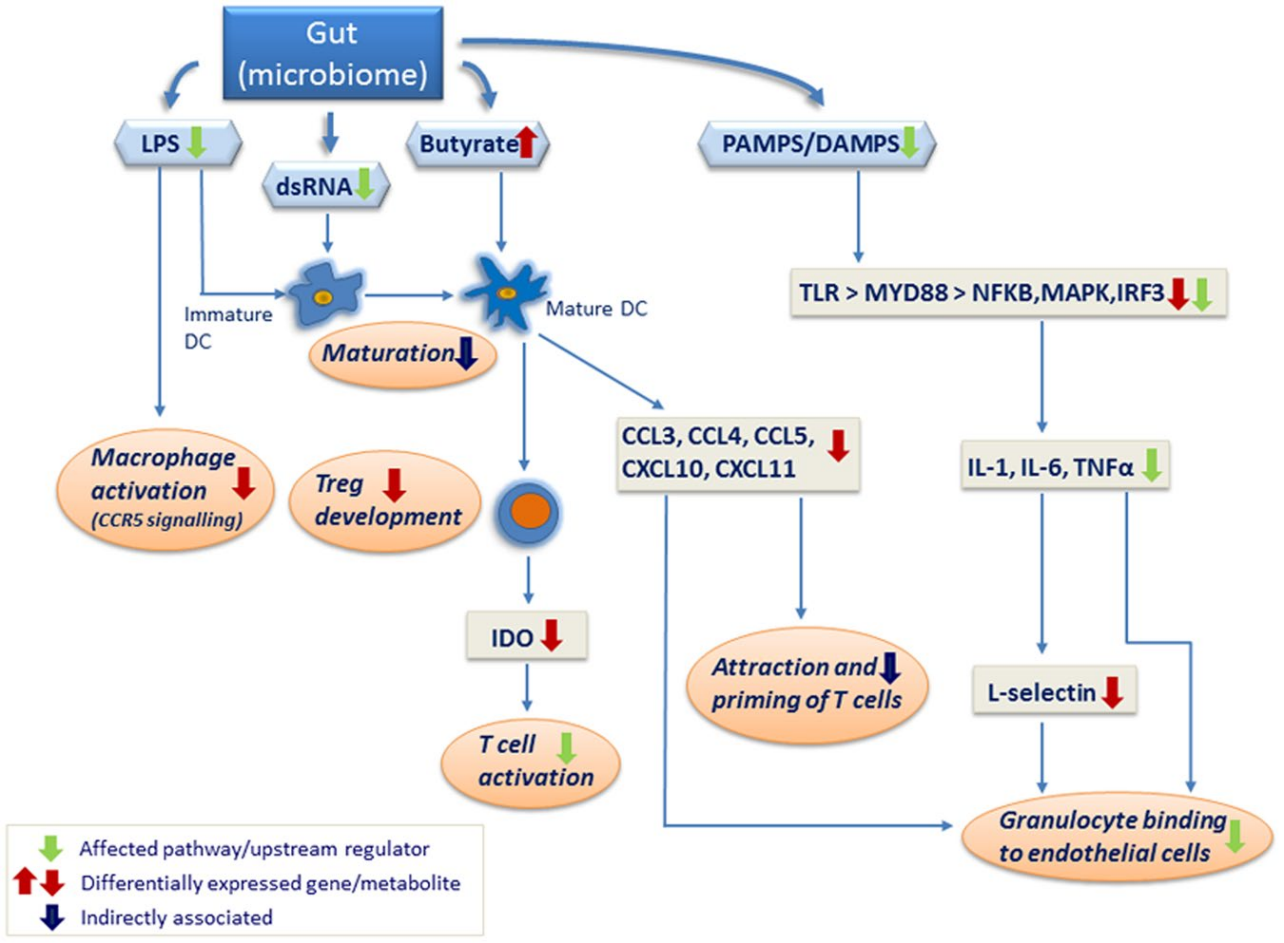
Model of the immune defence network in pre-weaning calf jejunum samples from individuals on the restricted milk (replacer) diet compared to calves on the *ad libitum* diet as determined by whole transcriptome sequencing. “Affected pathway/upstream regulator” indicates a significantly affected pathway/upstream regulator as determined by Ingenuity Pathway Analysis; “Differentially expressed gene/metabolite” indicates differential gene expression or differential metabolite concentration in the two groups as experimentally verified in the present study; “Indirectly associated” indicates that association of mechanisms has been confirmed in the literature. The direction of the arrows indicates up/downregulation, increased/decreased activity, and elevated/decreased concentration in calves on the restricted milk (replacer) diet compared to calves with *ad libitum* access to milk (replacer).

**Figure 3:**
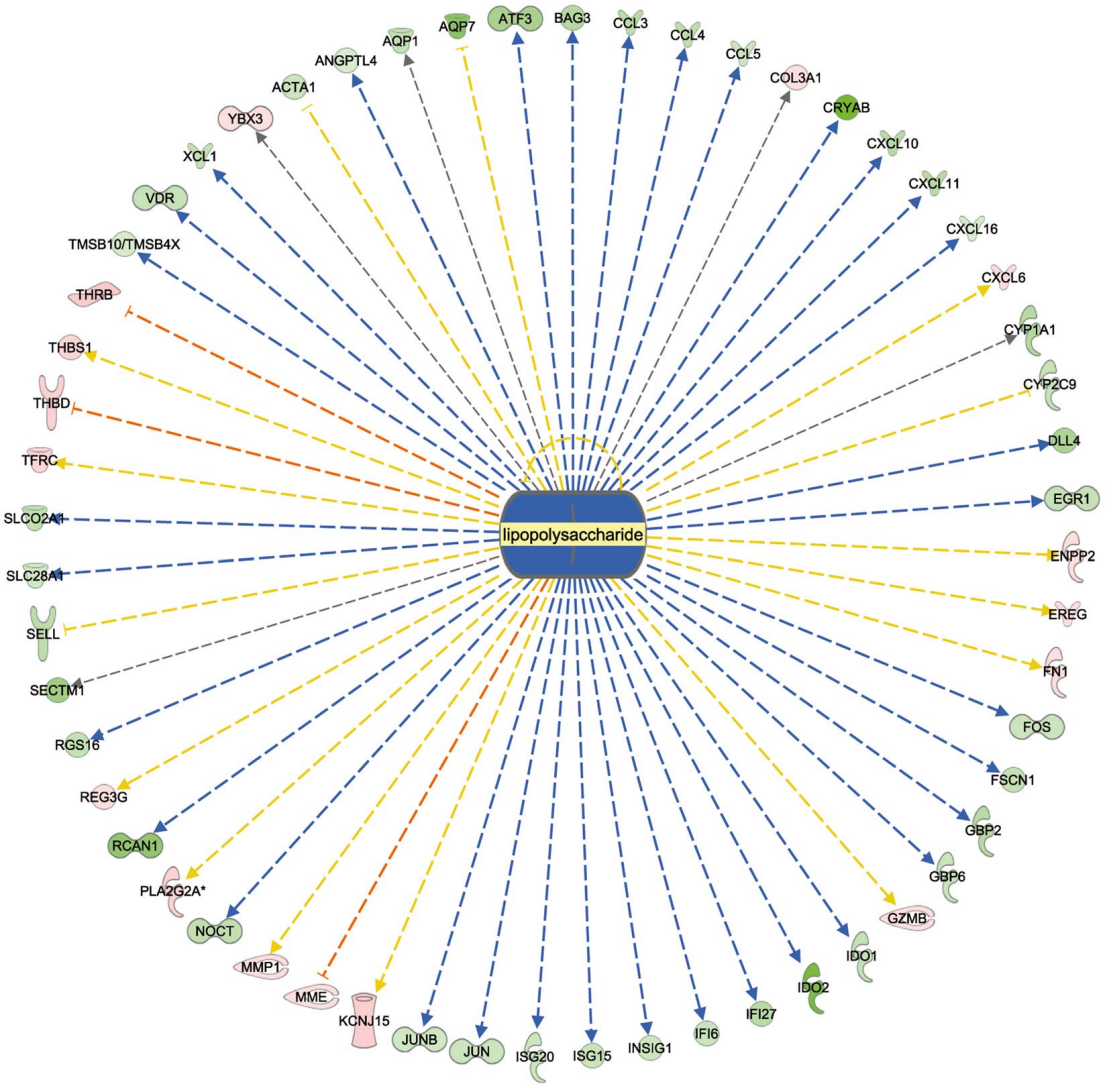
Confirmed downstream targets of lipopolysaccharide in the list of differentially expressed genes in pre-weaning calf jejunum samples from individuals on the restricted milk (replacer) diet compared to calves on the *ad libitum* milk (replacer) diet. Green indicates lower gene expression in calves on the restricted milk (replacer) diet compared to calves fed milk (replacer) *ad libitum;* red indicates higher gene expression in calves on the restricted milk (replacer) diet compared to calves fed milk (replacer) *ad libitum;* blue lines indicate predicted inactivation, brown lines indicate predicted activation, yellow lines indicate effects that are inconsistent with predictions, and grey lines indicate that no effect direction is predicted.

Our data can be clearly interpreted as indicating a coordinated lower activation state of the immune system in the RES calves compared to the AL animals (Fig. 2). This is further confirmed by the consistently decreased activation of immune response promoters, as indicated in the list of upstream regulators (see Table 5 and Fig. 1) in RES calves. A large number of molecules that modulate the immune response were identified as upstream regulators with a predicted inactivation status due to differentially decreased expression of their downstream targets. Potential exogenous drivers of inactivation could be viral or bacterial components, as suggested by the presence of LPS (Fig. 3) and poly rI:rC RNA (a synthetic dsRNA analogue) in the list of inactivated upstream regulators. Further evidence for inactivation of the immune system in the RES calves comes from the list of upstream regulators with predicted activation status; these regulators include the products of the *FOSL1*, *SOCS1* and *SOCS3*, *IRF4* and *ACKR2* genes, which are known for their inactivating effects on the immune system, e.g., through negative regulation of TLR signalling (*IRF4*)^29^ or for their major role in the immune silencing of macrophages (*ACKR2*)^30^.

**Figure 4:**
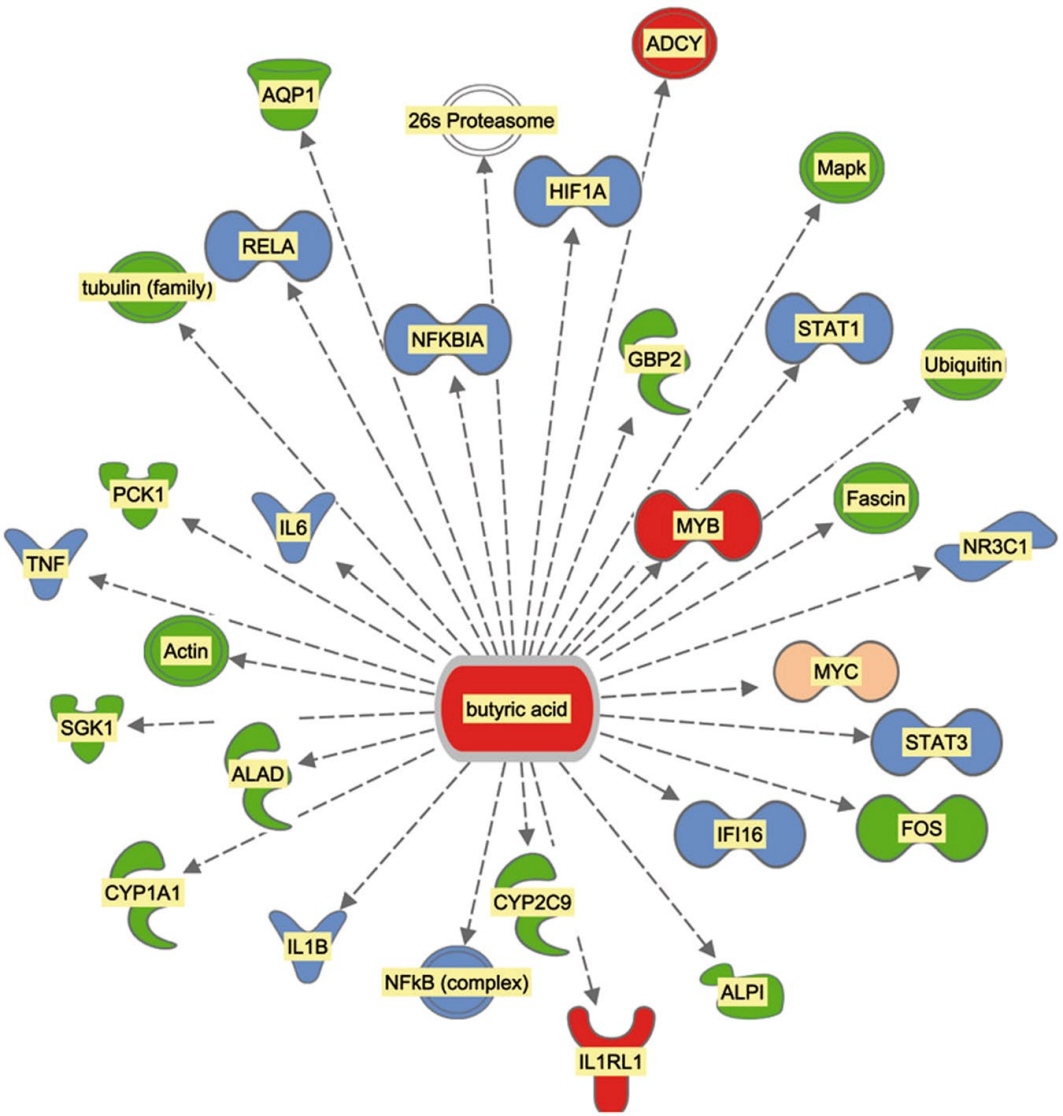
Confirmed targets of butyrate in the list of differentially expressed genes in pre-weaning calf jejunum samples from individuals on the restricted milk (replacer) diet compared to calves on the *ad libitum* diet. Red indicates differentially expressed genes/metabolites that were increased in calves on the restricted diet compared to calves on the *ad libitum* diet; green indicates differentially expressed genes/metabolites that were decreased in calves on the restricted diet compared to calves on the *ad libitum* diet; blue indicates significantly inactivated upstream regulators in calves on the restricted diet compared to calves on the *ad libitum* diet, and beige indicates significantly activated upstream regulators in calves on the restricted diet compared to calves on the *ad libitum* diet.

In addition to components of the microbial gut community (LPS, dsRNA), molecules originating from endogenous metabolism may also have affected the jejunal gene expression patterns of the two groups of calves, notably with respect to immune defence (Fig. 4). The coordinated lower transcript expression levels of genes encoding the small inducible cytokines CCL3, CCL4 and CCL5, which play a role in inflammatory responses, in the RES group compared to AL calves indicate a differential regulation of cytokine production in intestinal epithelial cells by interleukins IL-17A and IL-17F, which is demonstrated by a significant enrichment of the respective canonical pathway (Table 4). Together with the lower gene expression of *CXCL10* and *CXCL11*, both of which belong to the antimicrobial chemokine gene family, this coordinated regulation may reflect a reaction of mature dendritic cells to exposure to short-chain fatty acids, primarily butyrate, as was recently shown in an *in vitro* study by Nastasi *et al*.^31^.

From our gene expression data and data from the literature regarding the effects of butyrate on the immune system (e.g.,^31,32^), we propose a model in which butyrate has systemic effects on the jejunal cell population, predominantly the immune cells (Fig. 2). Systemic effects including immunomodulation of gut microbial-derived butyrate are well-known^33,34^, and starch microbial fermentation in the small intestine of calves had been confirmed^35^. Whether plasma β-hydroxybutyrate interferes in our suggested model is unclear at the moment and cannot be derived from the present study. Dendritic cells (DC) are important sentinel cells in mucosal tissues and represent a major component of the innate immune repertoire of mucosal tissues, including the bovine gut^36,37^. DC are matured by, e.g., LPS, which in our study is one of the most significant upstream regulators identified by IPA analysis (Table 5 and Fig. 3). The presence of mature DC in the jejunal mucosa of the calves in our data set is supported by the expression of *CD86* and *CD209*, typical markers for DC maturation. In their study, Nastasi and co-workers^31^ reported granulocyte adhesion and diapedesis as the most affected canonical pathway when *in vitro* matured DC were treated with butyrate. Granulocyte adhesion and diapedesis was also the most significantly affected canonical pathway identified from our data set comparing AL and RES (Table 4).

Specialized enteric DC promote the development of induced regulatory T cells (Treg), predominantly in the intestine. Arpaia and co-workers^38^ showed that butyrate enables the generation of Treg cells outside the thymus. In ruminants and specifically in calves, Treg cells are predominantly γ/δ T cells^26^. In our data set, the *TRGC3* gene, which encodes the T cell receptor gamma C3, displayed significantly elevated expression in AL calves compared to RES calves, fitting the hypothesis of the presence of an increased number of γ/δ Treg (Fig. 2; also see Supplementary Table S2). After maturation in cryptopatches of the intestine, Treg cells secrete IFNG, which subsequently results in macrophage stimulation. Treg cells and DC produce indoleamine 2,3-dioxygenase (IDO), an inhibitor of T cell activation, and thus promote peripheral tolerance via oxidative tryptophan degradation^39^. In our data set, *IDO1* and *IDO2*, both of which encode indoleamine 2,3-dioxygenase, were significantly more highly expressed in AL calves than in RES calves, an observation consistent with the hypothesis that the different diets affected T cell regulation and activation (Fig. 2; also see Supplementary Table S2).

The RES calves in our experiment displayed significantly higher β-hydroxybutyrate concentrations in blood plasma than AL calves from the age of 5 weeks onward (see^27^) except for the last week of the study, when they showed a lower β-hydroxybutyrate concentration^27^. Anti-inflammatory effects have been described not only for butyrate but also for β-hydroxybutyrate^40^. It is a particular feature of ruminants that the vast majority of the butyrate absorbed from the rumen is converted into ketones (mainly β-hydroxybutyrate and acetoacetate) by the ruminal epithelium prior to release into the portal circulation^41,42^. In our study, we observed that plasma β-hydroxybutyrate was elevated in the RES group during the period of divergent feeding but not at the end of the experiment when the feeding regime was identical. Perhaps there was an increased use of β-hydroxybutyrate as substrate to cover energy demands for the compensating body growth observed in RES calves at the end of the experimental period. The decreased expression of immune-related genes in RES calves might also have contributed to the increased feed conversion for weight gain during the period of compensating body growth.Whether elevated use of β-hydroxybutyrate and decreased expression of immune related genes both directly contribute to the compensating growth of RES calves at the end of the experiment or if there is a causal association between β-hydroxybutyrate and immune system cannot be unequivocally identified and should be investigated in future experiments.

**Figure 5:**
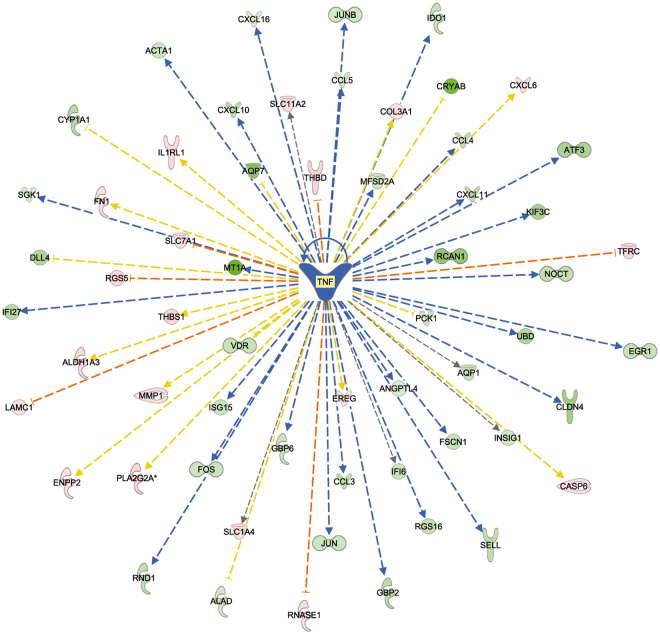
Differentially expressed genes in pre-weaning calf jejunum samples from individuals on the restricted milk (replacer) diet compared to calves on the *ad libitum* diet. The genes were confirmed to be regulated by TNF as upstream regulator. Green indicates lower gene expression in calves on the restricted milk (replacer) diet compared to calves fed milk (replacer) *ad libitum;* red indicates higher gene expression in calves on the restricted milk (replacer) diet compared to calves fed milk (replacer) *ad libitum;* blue lines indicate predicted activation, brown lines indicate predicted inactivation, yellow lines indicate effects that are inconsistent with predictions, and grey lines indicate that no effect direction is predicted.

In addition to the effects of diet on T cell activation via DC, our data also indicate an effect of diet on the innate TLR-MYD88-NFKB/MAPK/IRF3-regulated proinflammatory cytokines IL-1, IL-6 and TNFα. Considering the established regulation of SELL by TNF^43^, one result of the decreased activation of TNF in RES calves is presumably the reduced expression of *SELL*, the gene encoding L-selectin (Fig. 2). L-selectin plays a key role in granulocyte rolling and endothelial diapedesis^44^. Ballou and colleagues^23^ reported elevated TNF expression in leukocytes from AL calves after LPS stimulation compared to leukocytes from RES calves. Reduced TNF activity in the animals in the RES group is also indicated by the upstream regulator analysis of our data by IPA, which consistently showed lower expression levels of many genes known to be regulated by TNF (Fig. 5). However, in contrast to the results obtained by Ballou and colleagues in leukocytes sampled prior to weaning^23^, we observed increased expression of *SELL* in jejunal samples from AL calves at the end of the weaning process. Whereas the data from Ballou *et al*.^23^ are inconsistent (higher *TNF* but lower *SELL* expression), our data conclusively suggest that a post-weaning immunogenic stimulation occurs in the gastrointestinal tract of the AL calves, leading to higher immunological reactivity. It can be assumed that the granulocyte-associated immune response is decreased in RES calves compared to AL calves due to the direct effects of proinflammatory IL-1, IL-6 and TNFα on granulocyte binding to endothelial cells (Fig. 2). This conclusion is also supported by the higher expression of the genes encoding CCL3 and CCL4, which also act as neutrophil chemotactic cytokines^45^, in the AL group.

In the context of the intestinal immune response, another interesting gene that was differentially expressed in AL and RES calves is *ALPI*, which encodes an intestinal alkaline phosphatase. This enzyme plays an important role in the intestinal mucosal defence system and is effective in the neutralization of LPS and in preventing the translocation of bacteria^46,47^. The *ALPI* gene is expressed at a higher level in AL than in RES, suggesting a greater activation of intestinal defences against LPS challenge in AL animals.

In contrast to the highly significant results of the enrichment analyses regarding immune function, none of the enrichment data suggested that divergent metabolic responses occurred in the two groups. However, single groups of differentially expressed genes encoding proteins classified as enzymes, peptidases or transporters showed consistent differences in AL and RES calves. In general, consistently higher expression levels of amino acid transporters were observed in RES calves (Supplementary Fig. S5). *SLC1A4*, which encodes a sodium-dependent neutral amino acid transporter for alanine, serine, cysteine, and threonine, *SLC38A2*, which encodes the sodium-coupled neutral amino acid transporter 2, *SLC6A14*, which encodes the sodium- and chloride-dependent neutral and basic amino acid transporter B(0+), and *SLC7A1*, which encodes a cationic amino acid transporter, were all significantly more highly expressed in RES calves than in AL calves. Taken together, these data could be interpreted as indicating the presence of a greater number of amino acid transporters in the small intestines of RES than in AL calves. Total crude protein intake was greater in RES than in AL calves at the end of the study^27^ because milk replacer intake was more or less the same; however, concentrate intake by RES calves was greater. Substrate-mediated up-regulation of most of the amino acid transporters in the small intestine has been demonstrated^48^; therefore, amino acid transporter gene expression may have increased in RES calves at the end of the study due to their greater amino acid intake. Alternatively, the greater expression of amino acid transporters in RES calves could also be due to an increased need for amino acid transport on a per cell basis because tissue growth in these animals had not yet adapted to the nutrient intake.

The elevated gene expression of *PCK1* and *PDK2* indicated that decreased pyruvate oxidation and increased glucose synthesis occurred in the intestinal mucosa of AL calves. In a recent study, Schäff and colleagues^20^ reported elevated *PCK1* gene expression in the livers of *ad libitum* milk-replacer-fed calves compared to calves whose consumption of milk replacer was restricted. There is ongoing discussion regarding whether the intestine contributes to endogenous glucose production^49^. Nevertheless, the expression of *G6PC*, which encodes glucose-6-phosphatase, the final enzyme of the gluconeogenic pathway, in our data set as well as in the adult bovine^50^ is a strong indicator that gluconeogenic activity occurs in the bovine intestine. Because plasma glucose and insulin concentrations were lower in AL calves than in RES calves at the end of the study, there was probably a need for greater endogenous glucose production in AL calves to maintain glucose homeostasis in blood^27^. However, the relevance of endogenous glucose production by the bovine intestine has not been demonstrated, and this issue should be addressed in further investigations.

In both groups of calves, genes encoding proteins relevant to intestinal fatty acid metabolism (e.g., fatty acid-binding protein) were within the most highly expressed of all expressed genes in the small intestinal mucosa. For pre-ruminant calves, fat is an important source of energy for maintenance and growth, but fatty acids also contribute to the energy metabolism of the intestinal mucosa^51^. Fatty acid-binding proteins and apolipoproteins are involved in chylomicron formation and in the targeting of fatty acids within epithelial cells^52,53^. The high level of expression of genes encoding fatty acid-binding proteins in the jejunal epithelial cells of the calves in our study (Table 1) supports the importance of fatty acid-binding proteins in fatty acid metabolism in milk-fed calves^54^. In piglets, fatty acid metabolism contributes significantly to the energy metabolism of mature epithelial cells in the intestinal mucosa during weaning^55^. Our results indicate that the dominant and equal expression of genes related to fatty acid transport and apolipoprotein formation in the two groups may not depend on the level of milk intake during the pre-weaning period but instead may have resulted from feeding the same diet to both groups at the time of slaughter.

In conclusion, we observed that feeding of a restricted milk (replacer) diet instead of an *ad libitum* milk replacer diet in pre-weaned calves has substantial and prolonged effects on gene expression in the jejunal epithelium. In particular, calves fed an *ad libitum* diet exhibited very coordinated activated immune systems that were potentially modulated by the direct or indirect effects of butyrate and displayed decreased expression of amino acid transporters. Furthermore, several lines of evidence obtained in our study suggest that glucose metabolism was affected by changes in the neonatal diet. Particularly, the data on the divergent activity of the immune system in AL and RES calves suggest the importance of conducting further studies on the long-term health consequences of different early-life diets.

## Materials and Methods

### Animals, feeding, diets and performance of the calves

The animal experiment was conducted at the Educational and Research Centre for Animal Husbandry, Hofgut Neumuehle, Germany. In conformity with the German Animal Welfare Act, the trial (23 177-07/G 13-20-069) was permitted by the local department for animal welfare affairs (Landesuntersuchungsamt, Koblenz, Germany).

Twelve male German Holstein calves were reared from birth until 80.4 ± 1 days of age. Within two hours after birth, all calves received 2.5 ± 0.09 kg (mean ± SD) of colostrum from their dams through a bottle. Afterwards, the calves were allocated to one of the two feeding groups based on their birth weights and cow parity to create equal groups. For the following five meals (until the end of day 3 of age), the calves were fed acidified transition milk (2 mL acidifier/L milk, H. W. Schaumann GmbH, Pinneberg, Germany) from their dams with teat buckets either in amounts of 3 kg per meal (RES; n = 6) or *ad libitum* (AL; n = 6). From day 4 on, calves were fed a milk replacer (milk replacer; 125 g powder per L; Trouw Nutrition Deutschland GmbH, Burgheim, Germany) in amounts of either 6 L/d (RES) or *ad libitum* (max. 25 L/d; AL). The ingredients and the chemical composition of the milk replacer are given in Supplementary Table S6. The amount of milk replacer provided was stepped down linearly from day 57 to day 70 in all groups and was fed in amounts of 2 L/d until the end of the trial. During the first 10 ± 3 days (mean ± SD) of age, the feeding occurred twice a day at 07.00 and 17.00 h in individual hutches. For AL calves, the feeding buckets were kept with the calves between feeding times and were refilled if needed. After 10 days, feeding was conducted by automatic feeding systems for milk replacer and concentrate (Förster-Technik GmbH, Engen, Germany) in an open straw-bedded stable. Milk replacer was fed in small portions (a maximum of 3 L per meal for RES calves and a maximum of 5 L per meal for AL calves) followed by an off-time of 30 min after the end of the meal. Water was freely available, and hay and concentrate (Raiffeisen, Köln, Germany; see Supplementary Table S6) were offered *ad libitum* to all calves beginning at 10 ± 3 days of age.

Milk, milk replacer, and concentrate intake was documented daily from the first to the 11th week of age as previously described^27^. The nutrient compositions of the milk replacer and the concentrate were analysed by an accredited external laboratory (Landwirtschaftliche Untersuchungs- und Forschungsanstalt, Speyer, Germany) according to the Weender standard procedure^56^. Body weight was recorded weekly until the end of the trial using a mobile scale (Tru- Test Ltd., Auckland, New Zealand).

For analysis of blood plasma butyrate and plasma β-hydroxybutyrate, blood samples were collected at weekly intervals until day 77 after birth as previously described^27^. *Ad libitum* milk-fed calves were fasted for at least one hour prior to blood sampling. Blood from RES calves was drawn prior to the morning feeding in the calf hutches or after 1 h fasting in group pens. Analysis of butyrate in blood plasma and rumen was performed as recently described^57^.

### Phenotypic data analysis

Performance data are presented as LSM ± SE if not stated otherwise in the text and were evaluated by repeated-measures ANOVA using PROC MIXED in SAS for Windows (release 9.4; SAS Institute Inc., Cary, NC, USA, 2013). The ANOVA model included the fixed effects of feeding regimen (RES vs. AL), time, and their interaction. Repeated measures on each calf were considered using the repeated statement of the MIXED procedure with an unstructured type of block diagonal residual covariance matrix structure (SAS/STAT software, Version 9.3 of the SAS System for Windows; SAS Institute Inc.). Least squares means (LSM) and their standard errors (SE) were computed for each fixed effect in the models, and all pair-wise differences of LSM were tested using the Tukey-Kramer procedure. The SLICE statement of the MIXED procedure was used to conduct partitioned analyses of the LSM for interactions. Differences in data with P-values < 0.05 were defined as significant, and P-values < 0.1 were considered trends.

### Sampling and whole transcriptome sequencing

At day 80, the calves were slaughtered, and the abdominal cavity was opened immediately. The length of the small intestine was measured for each calf, and tissue samples were taken at the half length of the jejunum. The jejunum was defined according to a morphometric classification with the proximal end of the jejunum indicated by both the flexura duodenojejunalis and the cranial end of the plica duodenocolica and the distal end of the jejunum indicated by the plica ileocaecalis. The jejunum mucosa was collected by scraping the mucosa from the submucosa with a slide and was immediately snap-frozen in liquid nitrogen.

RNA from each sample was prepared using the NucleoSpin RNA kit (Macherey-Nagel, Düren, Germany). Quality control of samples comprised quantification via Qubit (Fisher Scientific) analysis, RNA integrity via Bioanalyzer (Agilent Genomics, Waldbronn, Germany) and testing of DNA contamination using a PCR test for a bovine genomic sequence according to Weikard and colleagues^58^. The obtained RNA samples had an average concentration of 823.2 ng/µl ( ±179.1) and an average RIN value after final DNA removal of 7.3 ( ±0.9). For each sample, an indexed, stranded sequencing library was prepared using the Illumina TruSeq Stranded mRNA library preparation kit (Illumina, San Diego, USA); preparation of the library included a polyA bead-based selection step. The libraries were sequenced using a 2 × 80 bp paired-end protocol on two lanes of an Illumina HiSeq. 2500 (Illumina, San Diego, USA). After demultiplexing, reads were trimmed for quality and adapter sequences with Cutadapt^59^ and in-house Linux tools. Reads that passed quality control were subjected to further bioinformatics analyses.

### Transcript assembly and analysis of differential expression

Essentially, analysis of differential transcript expression followed the workflow described by Trapnell and colleagues^60^. Initially, all samples were subjected to read alignment against the bovine reference genome assembly UMD3.1 (http://http.ensembl.org/././pub/release-83/fasta/bos_taurus/dna/) by the Bowtie/Tophat2 pipeline in a guided annotation approach using the Ensembl annotation 83 (http://http.ensembl.org/././pub/release-83/gtf/bos_taurus/) as the starting point. Subsequently, the aligned reads were assembled into contigs by Cufflinks2 in a guided approach, again using the Ensembl annotation 83 and the bovine reference genome assembly 3.1 as a starting point. The resulting gtf files from all samples and the Ensembl gtf file were merged by Cuffmerge to create the final gtf transcript annotation file, which served for transcript quantification via Cuffdiff2. We selected a guided assembly strategy because we knew from previous experiments that the current bovine genome annotation is rather incomplete and can lack highly significantly expressed transcripts. Cluster analysis, multidimensional scaling plots calculated from raw reads via edgeR^61^ and inspection of individual transcripts demonstrated that one sample was an outlier compared to all other samples. Inspection of the sample collection protocols indicated that for this sample no selective jejunal mucosa collection had been possible. Due to these issues, this sample was excluded from further analyses. Cuffdiff2 with pooled dispersion modelling was used to test for differential expression between calves fed *ad libitum* and calves fed the restricted diet. In parallel with Cuffdiff2 differential expression analysis, raw read counts as determined by Cuffdiff2 were used to calculate differences in transcript quantity between the two calf groups via edgeR^61^. The results of differential expression analyses were corrected for multiple testing^62^.

### Network and pathway analysis

For network and pathway analyses, all transcripts were considered, which had a gene symbol assignment in the bovine Ensembl 83 genome annotation and a q-value < 0.1 in the differential expression analysis between calves fed *ad libitum* and calves fed a restricted milk diet. Furthermore, for transcripts with q < 0.1 but with unknown annotation in Ensembl, we screened the respective genomic positions in the bovine NCBI annotation. We manually edited the respective gene names for the transcript if there was concordance in position and exon-intron structure as indicated by inspection of the BAM files in the Integrated Genome Viewer (IGV)^63^.

Enrichment analysis interrogating KEGG pathways (Kyoto Encyclopedia of Genes and Genomes (http://www.genome.jp/kegg/) and Gene Ontology (GO) terms (http://geneontology.org/page/download-ontology) was performed using the online tool DAVID (version 6.8beta, https://david.ncifcrf.gov/ ^64^). The official gene symbol served as identifier. Due to the better annotation of the human genome, the human gene list served as the background list; this is a stringent approach because it has to be assumed that not all genes on the background list are expressed in juvenile bovine gut.

Furthermore, we performed Ingenuity pathway analyses (IPA) (https://www.qiagenbioinformatics.com/products/ingenuity-pathway-analysis/) to identify canonical pathways, upstream regulators and networks associated with differences in the jejunal transcriptomes of calves fed the different diets. Twenty bovine gene symbols were not automatically recognized in the upload process and were edited manually to enable integration into the analysis.

### Availability of data

The data sets generated during and/or analysed during the study are accessible under the project number PRJEB24380 in the European Nucleotide Archive (ENA) (http://www.ebi.ac.uk/ena/submit).

## Electronic supplementary material

Supplementary Information
