# Supplementary material for: Different milk diets have substantial effects on the jejunal mucosal immune system of pre-weaning calves, as demonstrated by whole transcriptome sequencing
Source: Sci Rep. 2018 Jan 26;8:1693. doi: 10.1038/s41598-018-19954-2 (PMC5785999; doi:10.1038/s41598-018-19954-2)
Supplement: Supplementary file 1 — Supplementary Information [file 41598_2018_19954_MOESM1_ESM.pdf]

SUPPLEMENTARY INFORMATION

Different milk diets have substantial effects on the jejunal mucosal immune system of pre-weaning calves, as demonstrated by whole transcriptome sequencing

H.M. Hammon[1], D. Frieten[2], C. Gerbert[3], C. Koch[3], G. Dusel[2], R. Weikard[1], C. Kühn[1,4*]

[1] Leibniz Institute for Farm Animal Biology (FBN), Dummerstorf, Germany

[2] University of Applied Sciences, Bingen, Germany

[3] Educational and Research Centre for Animal Husbandry, Hofgut Neumühle, Münchweiler, Germany

[4] University Rostock, Faculty of Agricultural and Environmental Sciences, Rostock, Germany

[*]Corresponding author

Tel.: +49 38208 68709; fax: +49 38208 68702

Email: kuehn@fbn-dummerstorf.de

Experimental setup

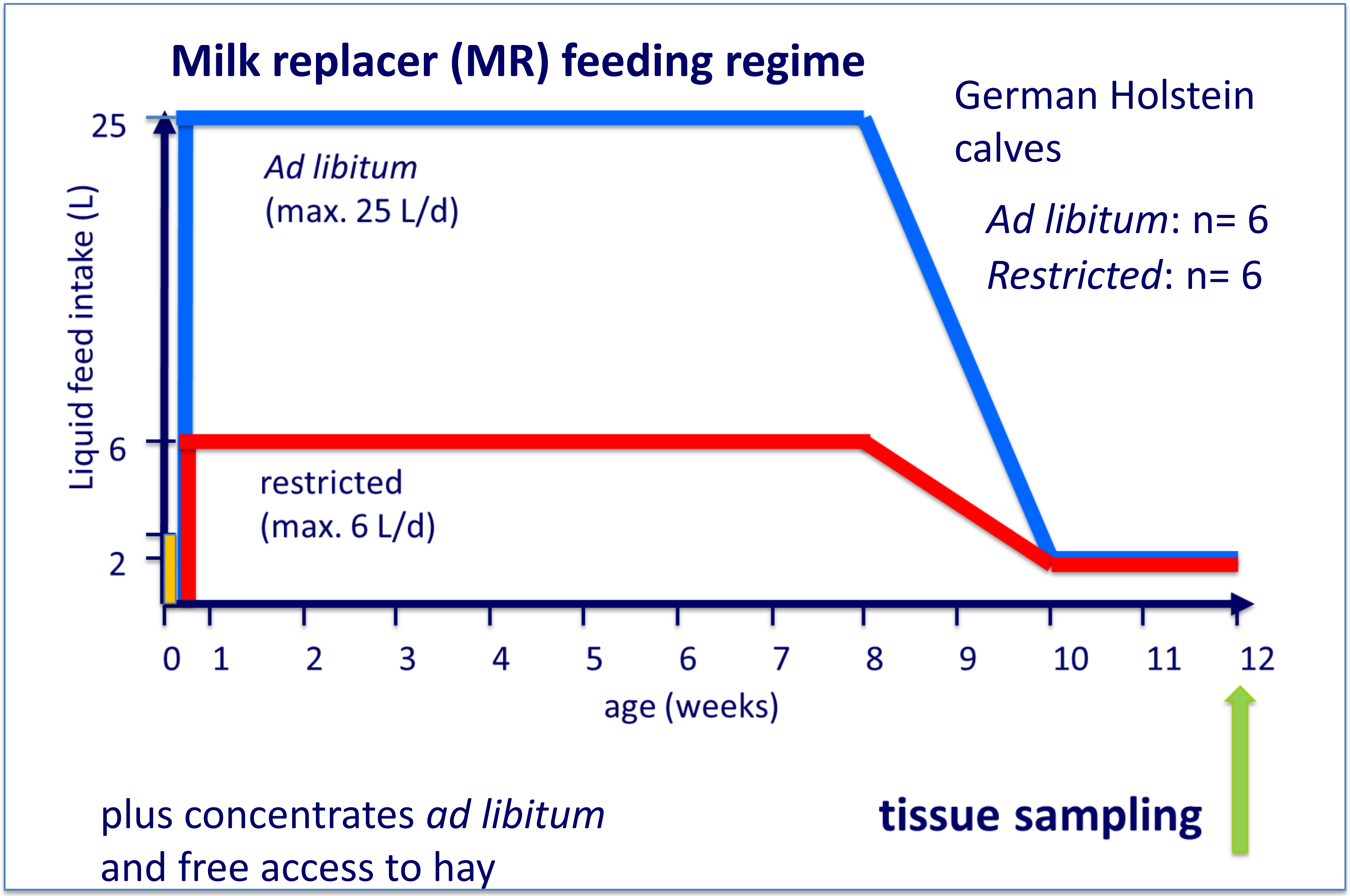

Supplementary Figure S1: Feeding regime for the two experimental groups of neonatal calves.
Blue: calves with unlimited access to milk (replacer), red: calves with restricted access to milk replacer. Hay and concentrate was provided ad libitum in both groups starting from day 10 after birth.

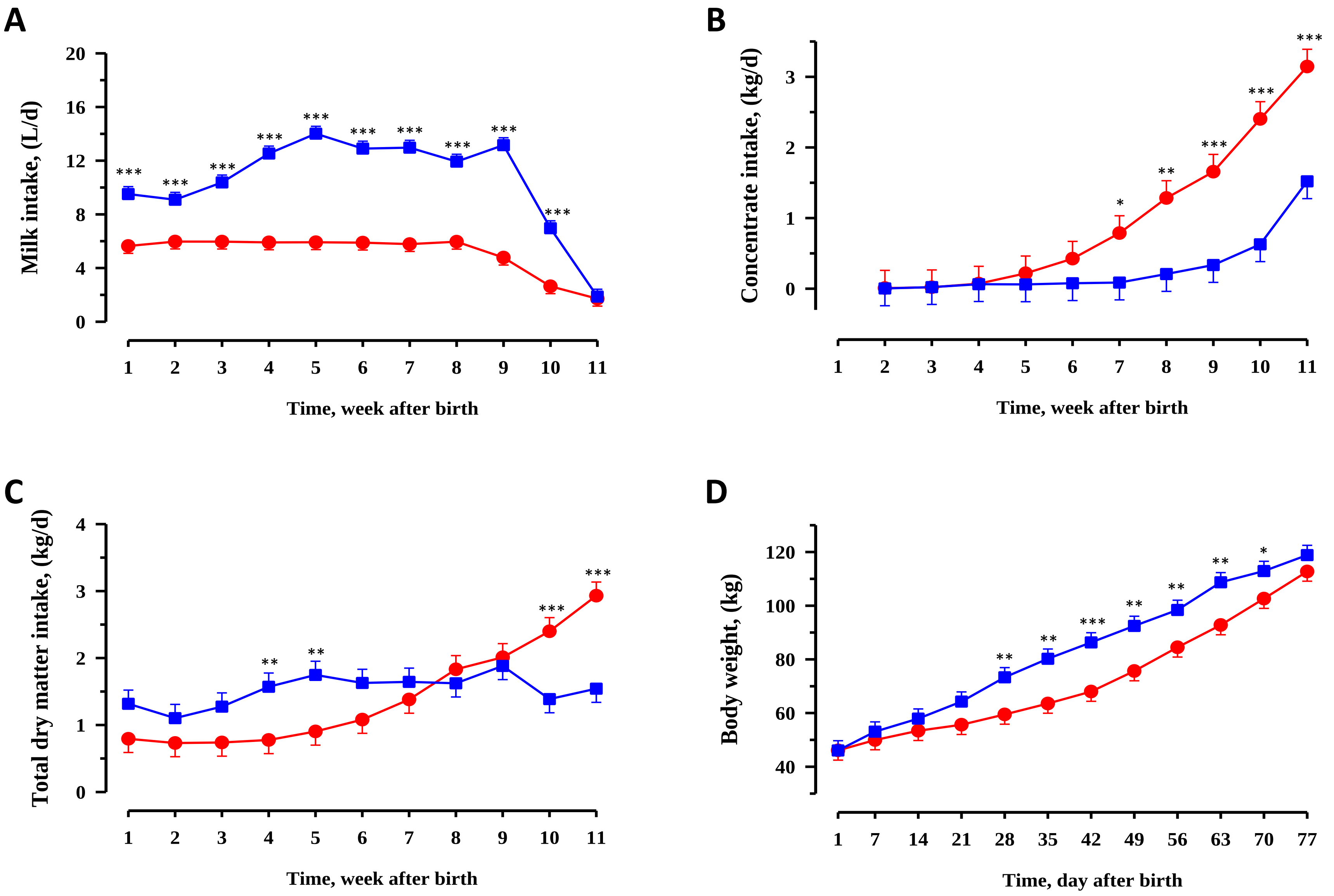

Supplementary Fig. S2: Milk (replacer), concentrate and dry matter intake and body weight of calves on an *ad libitum* (blue) or a restricted (red) milk (replacer) diet.
Asterix indicate statistical significance for difference between groups within week: *: $p<0.05$, **: $p<0.01$, ***: $p<0.001$

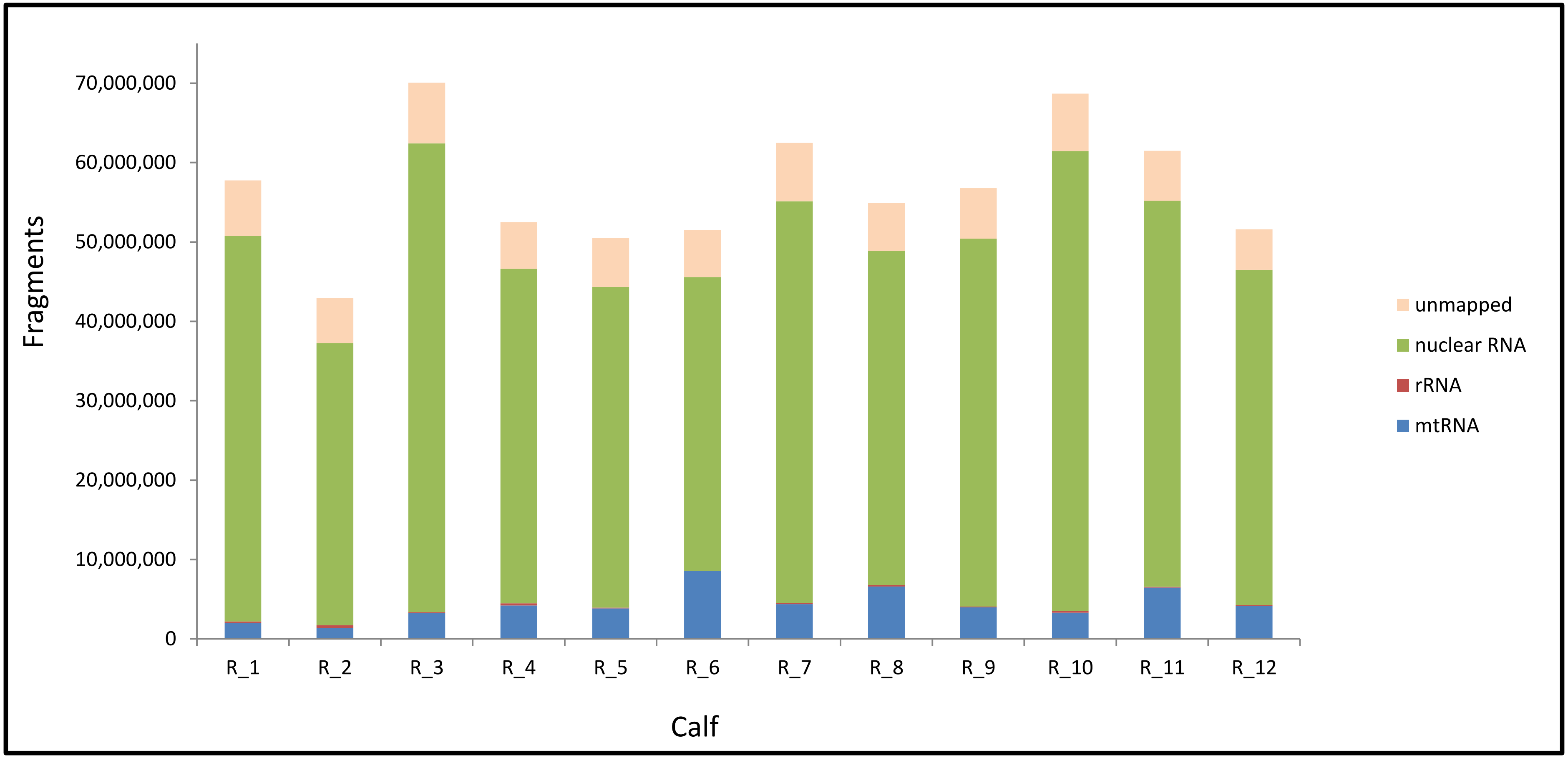

Supplementary Fig. S3: Fragments counts per class per calf from paired-end RNAseq data of jejunal tissue after quality trimming and alignment to the bovine reference assembly UMD3.1.
Classes: Unmapped: fragments with reads not mapped to the reference assembly, mtRNA: fragments with reads mapped to the bovine mitochondrial genome, rRNA: fragments with reads mapping to ribosomal RNA genes, nuclear_RNA: fragments with reads mapping to the nuclear (non-ribosomal RNA gene) genome. Individual calves indicated by R_...

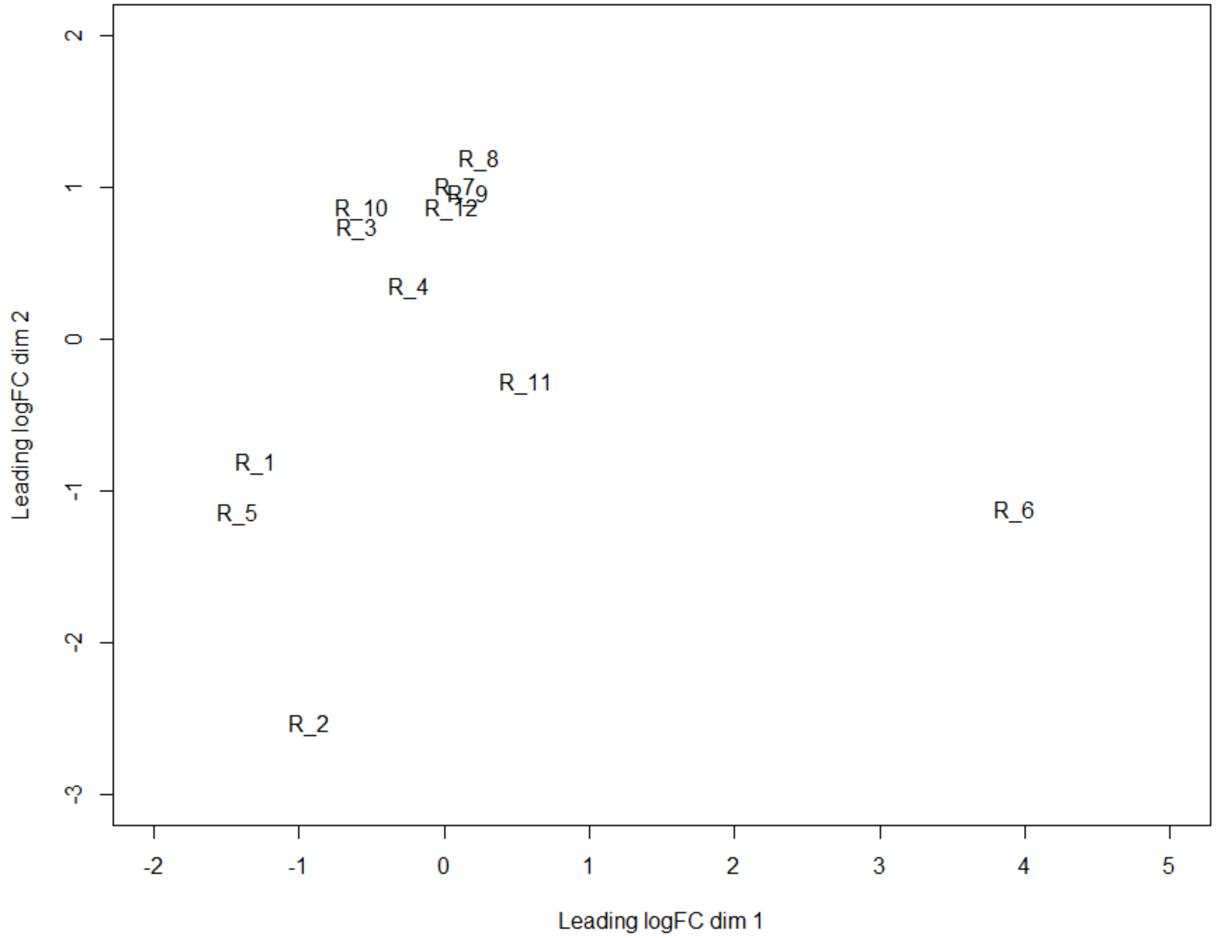

Supplementary Fig. S4: First two dimensions of a multidimensional scaling plot of read count data per locus per sample for samples from all calves initially included in the study.
R_.... indicates individual calves, logFC: log fold change between the groups fed either a restricted or ad libitum milk (replacer) diet

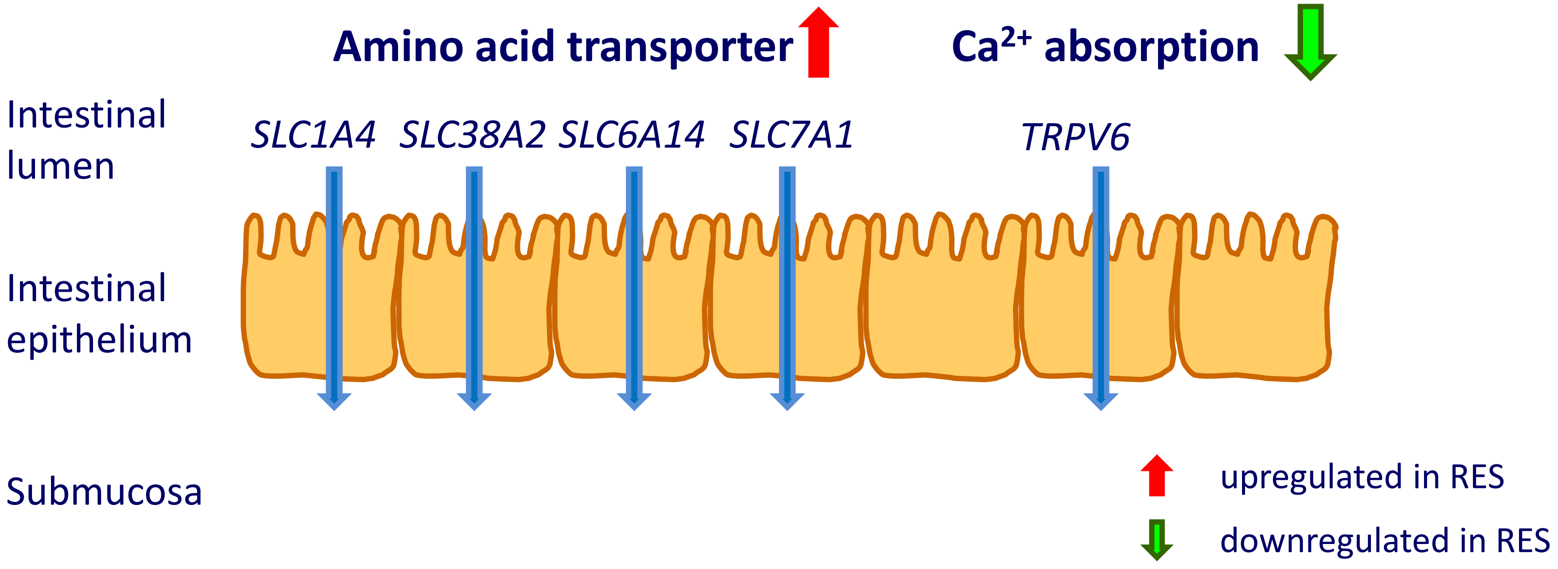

Supplementary Fig. S5: Significantly differentially expressed amino acid transporter genes and gene involved in Ca2+ absorption in the jejunal mucosa between calves on a restricted milk (replacer) diet (RES) compared to calves with *ad libitum* access to milk (replacer) (AL).

Supplementary Table S1A: The 100 most highly expressed genes (in FPKM) within the AL and the RES group as determined by cuffdiff read counting and normalization

Gene	Chromosomal position	FPKM_AL	status RES
FABP1	11:47787638-47795270	59472.3	OK
APOA1	15:27919930-28206538	42658.8	OK
FABP2	6:7103333-7226151	38178.7	OK
APOC3	15:27914679-27917264	33042.0	OK
B2M	10:104139089-104145312	17949.2	OK
S100G	X:134046107-134155813	17420.3	OK
APOA4	15:27906598-27909370	13636.9	OK
RBP2	1:130684904-130710118	11431.3	OK
BOLA,ENSBTAG00000005146,JSP.1	23:28329978-28513351	7681.7	OK
ENSBTAG00000022715 (DMBT1)	26:42782328-42813472	5839.3	HIDATA
PRAP1	26:25881751-25885322	5374.3	OK
ENSBTAG00000031834,ISG12(B)	21:59275186-59322006	4923.0	OK
CRIP1	21:71390599-71392110	3769.3	OK
TM4SF4	1:119603877-119631566	3613.1	OK
ADA	13:73750478-73773983	3396.8	OK
KRT20	19:41720415-41732043	2959.7	OK
MUC13	1:69934412-69962033	2879.2	OK
DBI	2:71561191-71566372	2875.5	OK
CHRNA10	15:52184589-52259540	2813.0	OK
TPT1	12:15518991-15522535	2770.5	OK
PCBP2,PRR13	5:26702878-26733390	2699.4	OK
ACTB	25:39343632-39347044	2524.7	OK
ANXA13	14:17734073-17791429	2405.7	OK
TMSB10	11:49933203-49934214	2361.4	OK
MYL6	5:57486016-57489133	2077.3	OK
TMSB4	X:140973701-140975767	1991.5	OK
KRT20	19:41720415-41732043	1985.6	OK
C18H19orf33	18:48337452-48338289	1968.8	OK
FTH1	29:41172508-41175112	1932.8	OK
CHRNA10	15:52184589-52259540	1917.1	OK

ACTG1	19:51868428-51871276	1901.1	OK
ENSBTAG00000012363	2:120910018-120913882	1831.6	OK
CLCA4	3:57643205-57674223	1779.3	OK
ITM2B	12:18114552-18139506	1766.0	OK
TM4SF20	2:116299720-116377650	1764.3	OK
LCT	2:61866684-61918264	1670.9	OK
UBB	19:33853784-33855991	1630.5	OK
FTL	18:55992909-55994741	1613.4	OK
RPLP1	10:16324340-16326492	1593.8	OK
CD74,RPS14	7:63748884-63779547	1553.0	OK
CCL25	7:17976645-18003965	1551.3	OK
RPS11	18:56404236-56406668	1548.8	OK
P4HB	19:51643764-51653754	1505.5	OK
RPL37	20:33667204-33669805	1449.8	OK
EEF1A1	9:13233553-13236949	1409.9	OK
GAPDH	5:104237901-104241979	1396.2	OK
RPS12	9:71974706-71978200	1395.9	OK
ATP1A1	3:27002621-27033781	1391.5	OK
ENPP3	9:70588606-70673279	1373.3	OK
KRT8	5:27213631-27221177	1371.9	OK
RPS24	28:33926417-33931777	1360.8	OK
RPL23	19:40074999-40079360	1360.4	OK
RPS27	3:16505731-16507247	1342.4	OK
ALDOB,TMEM246	8:92773691-92865307	1274.8	OK
ATOX1	7:64945748-64961110	1259.1	OK
SGLT1	17:72672983-72741764	1256.7	OK
SMIM24	7:21660936-21667474	1256.7	OK
TARS	20:40349624-40373064	1256.3	OK
PLA2G16	29:42454971-42608419	1245.9	OK
UBA52	7:4535466-4537851	1240.1	OK
ACSL5	26:33184422-33234956	1230.7	OK
ENSBTAG00000047694	21:55966631-55968362	1225.6	OK
VIL1	2:107145366-107255528	1176.2	OK

CFL1	29:44638895-44642280	1162.2	OK
RPL31	11:6003174-6008528	1143.7	OK
ENSBTAG00000033979	23:25797812-26178106	1123.3	OK
LGALS4	18:48803146-48810095	1122.2	OK
MGST3	3:3296281-3319165	1112.5	OK
ANXA2	10:49859972-49904536	1105.4	OK
BOLA,ENSBTAG00000001476,ENSBTAG00000007075,EN[illegible]	23:27646914-27871795	1095.2	OK
ENSBTAG00000025398,ENSBTAG00000031825	23:28813848-28875867	1091.9	OK
BCL2L15	3:29643175-29648016	1084.9	OK
MS4A8B	29:37636996-37654922	1081.3	OK
ENSBTAG00000046041	12:71213741-74032603	1064.2	OK
BCMO1	18:7922946-7967945	1033.3	OK
RPL27A	15:44469326-44472127	1020.7	OK
HSD17B2	18:8607690-8729527	1011.8	OK
LGALS3	10:67843327-67861113	997.9	OK
ENSBTAG00000047869,PHYH	13:28204599-28275573	991.1	OK
PFN1	19:27080355-27084643	975.0	OK
TXN	8:101484811-101504018	968.2	OK
COX4I1	18:11788218-11807341	937.9	OK
ENSBTAG00000015047	6:86676446-86749303	919.0	OK
ISG15	16:52714626-52715665	888.7	OK
RPS25	15:30107960-30117641	887.4	OK
IFI27	21:59325290-59336752	883.8	OK
RPS28	7:18198991-18200124	874.6	OK
GDE1	25:17210000-17237789	870.6	OK
LAPTM4B	14:68668785-68730708	868.5	OK
ATP1B3	1:128050717-128085713	850.8	OK
HSPA8	15:34216277-34220705	844.9	OK
CDH17	14:72436999-72525873	842.8	OK
COX6A1	17:64995247-64997121	842.8	OK
CES2	18:34757952-34770083	837.5	OK
RPS2	25:1520483-1522670	833.2	OK
RPS15A	25:16531760-16537583	831.7	OK

COX7C	7:88648419-88657334	825.6	OK
CHRNA10	15:52184589-52259540	824.5	OK
RPL13A	18:56394557-56398082	823.7	OK
S100A10	3:18799569-18811411	822.3	OK

status_RES: HIDATA: FPKM quantification failed in group AL due to number of reads exceeding a cuffdiff upper threshold
status_RES: OK: FPKM quantification successful in group AL

Supplementary Table S1B: The 100 most highly expressed genes (in FPKM) within the AL and the RES group as determined by cuffdiff read counting and normalization			
**Gene**	**Chromosomal position**	**FPKM_RES**	**status AL**
FABP1	11:47787638-47795270	50984.1	OK
FABP2	6:7103333-7226151	27546.1	OK
APOA1	15:27919930-28206538	17253.8	OK
S100G	X:134046107-134155813	15575.2	OK
APOC3	15:27914679-27917264	11620.8	OK
B2M	10:104139089-104145312	10641.7	OK
RBP2	1:130684904-130710118	8229.4	OK
MUC13	1:69934412-69962033	4964.1	OK
APOA4	15:27906598-27909370	4735.3	OK
BOLA,ENSBTAG00000005146,JSP.:	23:28329978-28513351	3988.0	OK
ADA	13:73750478-73773983	3170.8	OK
TM4SF4	1:119603877-119631566	3006.1	OK
KRT20	19:41720415-41732043	2794.3	OK
ENSBTAG00000031834,ISG12(B)	21:59275186-59322006	2722.2	OK
PRAP1	26:25881751-25885322	2567.9	OK
CHRNA10	15:52184589-52259540	2565.9	OK
TPT1	12:15518991-15522535	2334.4	OK
CRIP1	21:71390599-71392110	2059.5	OK
DBI	2:71561191-71566372	1943.7	OK
CHRNA10	15:52184589-52259540	1859.3	OK
KRT20	19:41720415-41732043	1801.9	OK
TMSB10	11:49933203-49934214	1785.0	OK
ACTB	25:39343632-39347044	1781.4	OK
TM4SF20	2:116299720-116377650	1740.1	OK
ANXA13	14:17734073-17791429	1686.1	OK
PIGR	16:4533504-4551642	1646.1	OK
MYL6	5:57486016-57489133	1557.3	OK
RPS12	9:71974706-71978200	1553.7	OK
EEF1A1	9:13233553-13236949	1550.5	OK
RPS11	18:56404236-56406668	1515.1	OK

ACTG1	19:51868428-51871276	1493.3	OK
RPS24	28:33926417-33931777	1486.8	OK
RPLP1	10:16324340-16326492	1486.1	OK
RPS27	3:16505731-16507247	1478.8	OK
PCBP2,PRR13	5:26702878-26733390	1454.0	OK
CD74,RPS14	7:63748884-63779547	1418.3	OK
RPL23	19:40074999-40079360	1409.3	OK
LCT	2:61866684-61918264	1407.2	OK
ATP1A1	3:27002621-27033781	1390.1	OK
GAPDH	5:104237901-104241979	1385.3	OK
RPL37	20:33667204-33669805	1353.3	OK
FTH1	29:41172508-41175112	1303.7	OK
FTL	18:55992909-55994741	1283.9	OK
LGALS4	18:48803146-48810095	1282.9	OK
KRT8	5:27213631-27221177	1249.9	OK
ENSBTAG00000046041	12:71213741-74032603	1244.4	OK
CCL25	7:17976645-18003965	1242.1	OK
P4HB	19:51643764-51653754	1230.7	OK
ITM2B	12:18114552-18139506	1214.2	OK
TARS	20:40349624-40373064	1199.0	OK
RPL27A	15:44469326-44472127	1140.1	OK
TMSB4	X:140973701-140975767	1126.7	OK
C18H19orf33	18:48337452-48338289	1121.4	OK
UBB	19:33853784-33855991	1117.8	OK
CLCA4	3:57643205-57674223	1114.7	OK
RPL31	11:6003174-6008528	1094.8	OK
SGLT1	17:72672983-72741764	1030.2	OK
MGST3	3:3296281-3319165	1014.8	OK
ENPP3	9:70588606-70673279	1009.9	OK
UBA52	7:4535466-4537851	994.7	OK
ENSBTAG00000012363	2:120910018-120913882	971.0	OK
ALDOB,TMEM246	8:92773691-92865307	960.6	OK
ENSBTAG00000047694	21:55966631-55968362	957.3	OK

SPINK4	8:76329290-76350361	939.7	OK
SMIM24	7:21660936-21667474	938.6	OK
ATOX1	7:64945748-64961110	935.5	OK
CFL1	29:44638895-44642280	926.4	OK
ATP6,COII,COX1,COX3,MT-ATP8,M	MT:0-16338	921.3	OK
RPS27A	11:37823233-37825489	918.3	OK
MS4A8B	29:37636996-37654922	916.9	OK
RPS25	15:30107960-30117641	912.3	OK
RPS8	3:101816843-101819459	907.0	OK
RPS15A	25:16531760-16537583	905.6	OK
PLA2G16	29:42454971-42608419	896.9	OK
COX4I1	18:11788218-11807341	893.5	OK
CHRNA10	15:52184589-52259540	889.7	OK
RPS2	25:1520483-1522670	888.1	OK
VIL1	2:107145366-107255528	887.8	OK
TXN	8:101484811-101504018	885.0	OK
ACSL5	26:33184422-33234956	872.9	OK
RPL39	X:97604640-97605063	862.6	OK
COX6A1	17:64995247-64997121	857.9	OK
RPL32	22:56985248-56989012	855.5	OK
ANXA2	10:49859972-49904536	854.3	OK
RPSA	22:12728209-12741653	846.6	OK
RPL10	X:40364801-40375771	829.6	OK
LGALS3	10:67843327-67861113	816.2	OK
COX7C	7:88648419-88657334	814.6	OK
RPS20	14:24955078-24956455	810.2	OK
ENSBTAG00000015047	6:86676446-86749303	797.9	OK
PRDX1	3:101152891-101166725	786.1	OK
AGR2	4:25258618-25271426	779.9	OK
ATP5B	5:57119916-57125290	767.7	OK
ENSBTAG00000046353,ENSBTAG(	3:113883858-114031371	761.5	OK
ATP5J2	25:37494138-37499276	759.8	OK
ENSBTAG00000039928	4:113775212-114087025	758.5	OK

RPL23A	19:20703987-20706890	751.9	OK
RPS17	21:23301662-23305104	751.4	OK
EPCAM	11:29626086-29636759	750.9	OK
ATP5A1	24:46300458-46309366	749.2	OK

status_AL: HIDATA: FPKM quantification failed in group AL due to number of reads exceeding a cuffdiff upper threshold
status_AL: OK: FPKM quantification successful in group AL

Supplementary Table S2: List of differentially expressed loci between calves fed a restricted or *ad libitum* milk replacer diet
AL: ad libitum group; RES: group with restricted milk replacer feeding

Gene_id	Updated_Gene list	Chromosomal position	FPKM_AL	FPKM_RES	$log2_{(fold\ change)}$	p_value	q_value
XLOC_028587	ADCY6	5:31160698-31176231	4.757	22.028	2.21119	5.00E-05	0.00880776
XLOC_015789	ALPI	2:120820818-120908355	400.722	141.385	-1.50298	5.00E-05	0.00880776
XLOC_033608	ANXA10	8:348902-544502	1.331	11.965	3.16826	5.00E-05	0.00880776
XLOC_023995	ANXA8	28:42288942-42305289	11.205	3.528	-1.66738	5.00E-05	0.00880776
XLOC_027995	AQP1	4:65830987-65845398	31.371	14.095	-1.15422	5.00E-05	0.00880776
XLOC_010317	ATF3	16:72820025-72832974	29.013	8.849	-1.71312	5.00E-05	0.00880776
XLOC_014762	B4GALNT2	19:38019225-38099045	0.122	57.733	8.8873	5.00E-05	0.00880776
XLOC_022669	BAG3	26:40118555-40141989	28.987	10.703	-1.43746	5.00E-05	0.00880776
XLOC_009296	C4BPA	16:4785223-4950977	23.293	60.344	1.37331	5.00E-05	0.00880776
XLOC_004810	C8G	11:106284926-10628703	77.829	22.921	-1.76366	5.00E-05	0.00880776
XLOC_033879	CA9	8:60247363-60266465	137.822	22.230	-2.63223	5.00E-05	0.00880776
XLOC_016946	CCDC152	20:31783961-31834092	321.409	133.503	-1.26754	5.00E-05	0.00880776
XLOC_021720	CLDN15	25:36040354-36045878	32.638	75.683	1.21343	5.00E-05	0.00880776
XLOC_022227	CLDN4	25:33988116-33989662	159.680	41.110	-1.95762	5.00E-05	0.00880776
XLOC_027454	CPA1	4:94943346-94950038	30.512	4.208	-2.85819	5.00E-05	0.00880776
XLOC_015645	CPO	2:95668824-95700853	47.271	7.762	-2.60643	5.00E-05	0.00880776
XLOC_008628	CRYAB	15:22566928-22570256	192.689	21.111	-3.19023	5.00E-05	0.00880776
XLOC_031303	CXCL10	6:92597071-92755993	55.997	21.514	-1.38006	5.00E-05	0.00880776
XLOC_031303	CXCL11	6:92597071-92755993	55.997	21.514	-1.38006	5.00E-05	0.00880776
XLOC_017640	CYP1A1	21:34339809-34346375	78.403	28.772	-1.44625	5.00E-05	0.00880776
XLOC_022485	CYP2C18	26:15931931-15973962	71.859	24.481	-1.55351	5.00E-05	0.00880776
XLOC_026814	CYP4B1	3:99937151-99957408	28.798	1.498	-4.26512	5.00E-05	0.00880776
XLOC_016233	DAPL1	2:37593289-37620037	47.427	125.238	1.40089	5.00E-05	0.00880776
XLOC_025619	DDAH1	3:58808969-58965541	4.031	10.972	1.44471	5.00E-05	0.00880776
XLOC_019687	DDAH2	23:27399246-27402833	18.727	6.921	-1.43596	5.00E-05	0.00880776
XLOC_001948	DLL4	10:36577137-36586367	6.692	2.183	-1.61638	5.00E-05	0.00880776
XLOC_032252	EGR1	7:51438726-51442500	11.881	5.506	-1.10961	5.00E-05	0.00880776
XLOC_022585	ENSBTAG00000003989	26:25088447-25097722	34.855	2.719	-3.68043	5.00E-05	0.00880776

XLOC_027342	ENSBTAG00000015749	4:74836196-74849247	13.609	32.536	1.25746	5.00E-05	0.00880776
XLOC_025912	ENSBTAG00000017892	3:106893915-106934190	24.655	5.832	-2.07978	5.00E-05	0.00880776
XLOC_034632	ENSBTAG00000020105	8:76466807-76484933	36.664	6.861	-2.41778	5.00E-05	0.00880776
XLOC_034119	ENSBTAG00000021991	8:91818631-91915666	0.069	0.559	3.0267	5.00E-05	0.00880776
XLOC_009296	ENSBTAG00000039196	16:4785223-4950977	23.293	60.344	1.37331	5.00E-05	0.00880776
XLOC_018050	ENSBTAG00000046611	21:9614225-9635376	30.106	10.082	-1.57831	5.00E-05	0.00880776
XLOC_036319	ENSBTAG00000047547	X:94701433-94701912	16.174	53.127	1.71581	5.00E-05	0.00880776
XLOC_010092	ESPN	16:47756456-47791502	106.692	53.862	-0.986114	5.00E-05	0.00880776
XLOC_030076	GABARAPL1	5:100205185-100215034	315.472	143.062	-1.14087	5.00E-05	0.00880776
XLOC_026061	GAL3ST2	3:121247597-121267893	0.048	0.491	3.34659	5.00E-05	0.00880776
XLOC_025569	GBP2	3:54345400-54426844	76.687	29.302	-1.38797	5.00E-05	0.00880776
XLOC_013766	GIP	19:38202523-38207784	30.016	80.399	1.42146	5.00E-05	0.00880776
XLOC_025875	GUCA2A	3:104603592-104605588	815.249	351.710	-1.21285	5.00E-05	0.00880776
XLOC_001094	HEG1	1:70022649-70106415	9.908	4.412	-1.16711	5.00E-05	0.00880776
XLOC_023234	IDO2	27:34707852-34783680	22.605	2.881	-2.97184	5.00E-05	0.00880776
XLOC_017766	IFI27	21:59325290-59336752	883.778	338.349	-1.38517	5.00E-05	0.00880776
XLOC_015869	IFI6	2:126246550-126250182	221.181	108.716	-1.02465	5.00E-05	0.00880776
XLOC_003292	IL1RL1	11:7097067-7141323	1.366	4.086	1.58079	5.00E-05	0.00880776
XLOC_005312	KBTBD7	12:11439369-11444109	25.200	5.530	-2.18799	5.00E-05	0.00880776
XLOC_020154	KCNK17	23:13160202-13173045	23.184	7.010	-1.7256	5.00E-05	0.00880776
XLOC_003691	KIF3C	11:73451144-73487055	1.869	0.548	-1.76947	5.00E-05	0.00880776
XLOC_004453	LBH	11:69592078-69618485	52.638	14.024	-1.90824	5.00E-05	0.00880776
XLOC_032359	LOC100296105	7:61417710-61421981	3.330	18.115	2.44352	5.00E-05	0.00880776
XLOC_030150	LOC100847453	5:104398821-104402665	9.470	3.451	-1.45617	5.00E-05	0.00880776
XLOC_004526	LOC101902225	11:77900485-77936781	8.979	0.553	-4.02131	5.00E-05	0.00880776
XLOC_009512	LOC101906383	16:46076613-46098065	16.445	4.909	-1.74408	5.00E-05	0.00880776
XLOC_027372	LOC104972148	4:77906094-77910010	22.573	6.414	-1.81521	5.00E-05	0.00880776
XLOC_009296	LOC506707	16:4785223-4950977	23.293	60.344	1.37331	5.00E-05	0.00880776
XLOC_020402	LOC786987	23:28813848-28875867	1091.910	302.669	-1.85104	5.00E-05	0.00880776
XLOC_027830	MACC1	4:28833730-28931566	0.111	0.723	2.69968	5.00E-05	0.00880776
XLOC_026939	MAP7D1	3:110233081-110254774	10.023	4.352	-1.20368	5.00E-05	0.00880776
XLOC_034859	MEGF9	8:111914763-111995270	12.675	4.036	-1.65102	5.00E-05	0.00880776
XLOC_026919	MFSD2	3:106724605-106737453	119.786	59.647	-1.00593	5.00E-05	0.00880776

XLOC_021384	MMP25	25:2478688-2521328	704.212	239.167	-1.55799	5.00E-05	0.00880776
XLOC_026165	MPTX	3:10579368-10582728	28.520	173.931	2.60846	5.00E-05	0.00880776
XLOC_012588	MT1A	18:24106721-24108521	11.133	0.821	-3.76047	5.00E-05	0.00880776
XLOC_035182	MYB	9:74222881-74258205	1.213	3.880	1.6771	5.00E-05	0.00880776
XLOC_005304	OLFM4	12:10667334-10690305	0.749	7.195	3.26363	5.00E-05	0.00880776
XLOC_015991	PLA2G2A	2:133289294-133295334	43.580	152.762	1.80956	5.00E-05	0.00880776
XLOC_004464	PLB1	11:71116626-71242833	499.122	152.541	-1.7102	5.00E-05	0.00880776
XLOC_014677	PMP22	19:33357140-33382707	231.438	42.262	-2.4532	5.00E-05	0.00880776
XLOC_006872	PTI	13:74867647-75021147	60.771	175.888	1.53321	5.00E-05	0.00880776
XLOC_024136	PTPN5	29:26244553-26303070	9.436	2.168	-2.12211	5.00E-05	0.00880776
XLOC_024102	RAB30	29:12658738-12762276	26.799	7.804	-1.7799	5.00E-05	0.00880776
XLOC_000004	RCAN1	1:242452-362911	93.939	20.132	-2.22225	5.00E-05	0.00880776
XLOC_006085	REM1	13:61619540-61628687	14.952	2.459	-2.60401	5.00E-05	0.00880776
XLOC_010258	RGS16	16:65127301-65134065	11.325	5.088	-1.15443	5.00E-05	0.00880776
XLOC_025039	RGS5	3:6228348-6428206	6.079	19.417	1.67537	5.00E-05	0.00880776
XLOC_024410	RHOD	29:45685401-45694819	69.194	26.811	-1.36781	5.00E-05	0.00880776
XLOC_010683	SDS	17:63302932-63311175	21.442	59.418	1.47044	5.00E-05	0.00880776
XLOC_014017	SECTM1	19:50867136-51149867	191.362	50.310	-1.92739	5.00E-05	0.00880776
XLOC_017771	SERPINA5	21:59782728-59795819	232.521	102.583	-1.18057	5.00E-05	0.00880776
XLOC_008498	SERPING1	15:82158388-82172146	249.624	87.672	-1.50957	5.00E-05	0.00880776
XLOC_036569	SLC6A14	X:730945-759703	7.324	17.821	1.28284	5.00E-05	0.00880776
XLOC_027245	STARD3NL	4:50267384-50328618	34.639	11.267	-1.62026	5.00E-05	0.00880776
XLOC_001109	TFRC	1:71257181-71331970	5.284	16.744	1.66395	5.00E-05	0.00880776
XLOC_023278	THRB	27:41335037-41776292	0.195	0.833	2.09292	5.00E-05	0.00880776
XLOC_024390	TMEM151A	29:44992204-44995718	13.770	4.323	-1.67121	5.00E-05	0.00880776
XLOC_014518	TRPV3	19:24850762-24885437	0.843	0.268	-1.65204	5.00E-05	0.00880776
XLOC_008316	TSKU	15:56961319-56974713	111.322	49.238	-1.17689	5.00E-05	0.00880776
XLOC_031870	TUBB4A	7:19206049-19215573	399.501	190.280	-1.07007	5.00E-05	0.00880776
XLOC_019762	UBD	23:28918427-28920742	500.708	163.119	-1.61805	5.00E-05	0.00880776
XLOC_020576	Unknown	23:40796533-40817043	1.742	0.209	-3.05797	5.00E-05	0.00880776
XLOC_023241	Unknown	27:36186469-36188702	1.385	0.239	-2.53274	5.00E-05	0.00880776
XLOC_025570	Unknown	3:54428570-54429964	12.546	3.802	-1.72251	5.00E-05	0.00880776
XLOC_033141	Unknown	7:39374932-39376419	23.511	6.767	-1.79676	5.00E-05	0.00880776

XLOC_036438	Unknown	X:124115248-124122231	1.876	0.124	-3.92315	5.00E-05	0.00880776
XLOC_003054	Unknown	10:79645279-79645726	0.000	0.273	inf	5.00E-05	0.00880776
XLOC_034898	Unknown	9:2184756-2185105	0.000	0.369	inf	5.00E-05	0.00880776
XLOC_010439	Unknown	17:9552021-9552152	0.000	9.365	inf	5.00E-05	0.00880776
XLOC_037126	Unknown	X:100211811-100211925	0.000	48.817	inf	5.00E-05	0.00880776
XLOC_032135	Unknown	7:43876075-43876201	0.000	72.074	inf	5.00E-05	0.00880776
XLOC_017065	Unknown	20:69966791-69966897	0.000	78.666	inf	5.00E-05	0.00880776
XLOC_034828	Unknown	8:104090079-104090215	0.000	156.166	inf	5.00E-05	0.00880776
XLOC_018636	Unknown	22:22308543-22308648	0.000	220.774	inf	5.00E-05	0.00880776
XLOC_031055	Unknown	6:28913574-28913665	0.000	301.286	inf	5.00E-05	0.00880776
XLOC_011246	Unknown	17:67217518-67217626	0.000	332.403	inf	5.00E-05	0.00880776
XLOC_026393	Unknown	3:25813283-25813367	0.000	418.861	inf	5.00E-05	0.00880776
XLOC_023200	Unknown	27:28003521-28003605	0.000	2068.620	inf	5.00E-05	0.00880776
XLOC_026448	Unknown	3:32722118-32722201	0.000	3142.750	inf	5.00E-05	0.00880776
XLOC_001660	Unknown	10:9699105-9700731	0.373	0.000	inf	5.00E-05	0.00880776
XLOC_001661	Unknown	10:9700814-9702130	0.487	0.000	inf	5.00E-05	0.00880776
XLOC_029728	Unknown	5:56187306-56187923	0.779	0.000	inf	5.00E-05	0.00880776
XLOC_011900	Unknown	18:50376456-50376939	1.559	0.000	inf	5.00E-05	0.00880776
XLOC_034121	Unknown	8:91975852-91978764	2.943	0.000	inf	5.00E-05	0.00880776
XLOC_002653	Unknown	10:24511998-24512565	3.149	0.000	inf	5.00E-05	0.00880776
XLOC_007323	Unknown	14:72577998-72578236	7.248	0.000	inf	5.00E-05	0.00880776
XLOC_009518	Unknown	16:46810269-46810386	16.515	0.000	inf	5.00E-05	0.00880776
XLOC_030576	Unknown	6:51332290-51332407	23.066	0.000	inf	5.00E-05	0.00880776
XLOC_006113	Unknown	13:63125998-63126109	37.289	0.000	inf	5.00E-05	0.00880776
XLOC_000455	Unknown	1:93776464-93776562	875.706	0.000	inf	5.00E-05	0.00880776
XLOC_024258	CD6	29:37892414-37938482	11.761	5.027	-1.22625	0.0001	0.015025
XLOC_009412	ENSBTAG00000009943	16:36963320-36966885	93.376	41.081	-1.18459	0.0001	0.015025
XLOC_021640	ENSBTAG00000014448	25:28101572-28161587	45.205	23.100	-0.968573	0.0001	0.015025
XLOC_026921	ENSBTAG00000046807	3:107133802-107174060	2.616	0.294	-3.15214	0.0001	0.015025
XLOC_023233	IDO1	27:34686487-34699492	138.462	63.108	-1.13358	0.0001	0.015025
XLOC_015931	IL22RA1	2:129351618-129378160	115.457	61.174	-0.916373	0.0001	0.015025
XLOC_010158	ISG15	16:52714626-52715665	888.741	423.798	-1.06838	0.0001	0.015025
XLOC_025720	JUN	3:87840170-87843838	45.479	22.702	-1.0024	0.0001	0.015025

XLOC_004219	KCNG3	11:24894832-24962486	0.201	0.634	1.66049	0.0001	0.015025
XLOC_000827	KCNJ15	1:152249959-152309628	0.461	1.900	2.04228	0.0001	0.015025
XLOC_009790	LOC519127	16:77795650-77829561	23.783	47.672	1.00321	0.0001	0.015025
XLOC_006142	MYH7B	13:64888625-64912866	2.638	0.912	-1.53215	0.0001	0.015025
XLOC_001935	PHGR1	10:36083908-36092734	162.617	77.970	-1.06048	0.0001	0.015025
XLOC_017280	RAI14	20:39351371-39515190	17.506	7.838	-1.1592	0.0001	0.015025
XLOC_017532	SLC28A1	21:22949799-23048041	111.096	59.370	-0.903998	0.0001	0.015025
XLOC_009858	SLC45A3	16:3243334-3262309	1.203	3.936	1.71016	0.0001	0.015025
XLOC_014450	TMIGD1	19:21960821-21976201	15.890	5.209	-1.60914	0.0001	0.015025
XLOC_018181	TRPM1	21:27992053-28061811	2.088	0.669	-1.64162	0.0001	0.015025
XLOC_032527	Unknown	7:100419543-100424322	0.195	0.974	2.32202	0.0001	0.015025
XLOC_036511	Unknown	X:136490614-136490886	0.000	0.352	inf	0.0001	0.015025
XLOC_027806	AGMO	4:23499625-23891372	25.980	10.589	-1.29489	0.00015	0.020434
XLOC_034838	ALAD	8:104342904-104352825	100.697	54.964	-0.873481	0.00015	0.020434
XLOC_001949	CHAC1	10:36598393-36600764	1.622	6.391	1.97868	0.00015	0.020434
XLOC_002328	ENSBTAG00000040078	10:87160345-87202080	54.402	25.881	-1.07175	0.00015	0.020434
XLOC_030746	EREG	6:91054423-91073724	4.352	10.965	1.33298	0.00015	0.020434
XLOC_007441	GRINA	14:2018543-2021709	65.701	35.262	-0.89782	0.00015	0.020434
XLOC_027690	INSIG1	4:117906797-117920029	134.719	68.564	-0.974425	0.00015	0.020434
XLOC_032821	JUNB	7:13851130-13852933	33.830	17.632	-0.940111	0.00015	0.020434
XLOC_008959	KCNE3	15:54584929-54598494	1.498	4.429	1.56373	0.00015	0.020434
XLOC_004226	LOC104973320	11:25480054-25559973	5.356	1.337	-2.00203	0.00015	0.020434
XLOC_017619	PSTPIP1	21:32644739-32689061	26.874	11.214	-1.26089	0.00015	0.020434
XLOC_036260	RGN	X:90730723-90747294	143.376	62.783	-1.19136	0.00015	0.020434
XLOC_034214	Unknown	8:112023788-112032429	5.791	2.331	-1.3127	0.00015	0.020434
XLOC_036419	Unknown	X:114171052-114171184	78.585	314.921	2.00267	0.00015	0.020434
XLOC_023778	ACTA1	28:419153-421910	61.099	30.573	-0.998898	0.0002	0.0261974
XLOC_014737	CACNA1G	19:36731339-36792963	2.007	0.536	-1.90542	0.0002	0.0261974
XLOC_014325	CCL4	19:14653466-14655028	25.644	10.609	-1.27327	0.0002	0.0261974
XLOC_013407	CCL5	19:14823469-14831157	396.797	203.026	-0.966736	0.0002	0.0261974
XLOC_002323	FOS	10:86883738-86887169	83.507	40.328	-1.05011	0.0002	0.0261974
XLOC_028610	VDR	5:32550494-32609211	25.885	12.142	-1.09208	0.0002	0.0261974
XLOC_016950	C6	20:33320060-33405570	6.651	13.300	0.999793	0.00025	0.0305898

XLOC_014019	CD7	19:51152074-51157233	57.619	25.746	-1.1622	0.00025	0.0305898
XLOC_013754	DLX3	19:37294964-37300157	1.855	0.520	-1.83493	0.00025	0.0305898
XLOC_007801	ENPP2	14:83360210-83499121	6.812	15.234	1.16118	0.00025	0.0305898
XLOC_026900	GUCA2B	3:104608980-104612513	415.044	215.073	-0.948437	0.00025	0.0305898
XLOC_009687	LAMC1	16:65545496-65665384	2.009	3.922	0.965229	0.00025	0.0305898
XLOC_030344	MAPK11	5:119830888-119837103	3.489	1.000	-1.80244	0.00025	0.0305898
XLOC_006689	PCK1	13:59144593-59150737	134.742	62.897	-1.09913	0.00025	0.0305898
XLOC_003597	REG3G	11:56528810-56531769	43.429	97.917	1.17291	0.00025	0.0305898
XLOC_010000	SELL	16:38147612-38173247	10.628	4.356	-1.28671	0.00025	0.0305898
XLOC_021845	ZFAND2A	25:42221232-42229176	13.527	4.631	-1.54646	0.00025	0.0305898
XLOC_022202	AUTS2	25:30043769-30153368	1.162	2.603	1.16311	0.0003	0.0356407
XLOC_001333	MME	1:113387230-113502881	3.580	8.162	1.18912	0.0003	0.0356407
XLOC_027365	NPC1L1	4:77565063-77597328	79.262	41.973	-0.91716	0.0003	0.0356407
XLOC_000683	SLCO2A1	1:136435428-136531202	19.598	8.428	-1.21742	0.0003	0.0356407
XLOC_009767	Unknown	16:75549862-75552455	0.465	0.082	-2.50422	0.0003	0.0356407
XLOC_022357	FSCN1	25:39292713-39302192	13.016	5.810	-1.16365	0.00035	0.0397328
XLOC_017469	ISG20	21:20013718-20031094	130.997	67.117	-0.964785	0.00035	0.0397328
XLOC_012590	MT1E	18:24117440-24118787	17.609	4.212	-2.06357	0.00035	0.0397328
XLOC_022660	PNLIPRP2	26:37346001-37366184	0.590	2.747	2.22035	0.00035	0.0397328
XLOC_035606	SGK1	9:73305299-73333706	88.712	46.702	-0.925658	0.00035	0.0397328
XLOC_006611	TGM3	13:53261634-53304213	0.148	0.670	2.17578	0.00035	0.0397328
XLOC_027396	TRGC3	4:83340026-83478290	82.248	35.845	-1.19821	0.00035	0.0397328
XLOC_027609	Unknown	4:109688457-109689622	170.164	0.062	-11.4317	0.00035	0.0397328
XLOC_018033	ALDH1A3	21:5793941-5833339	1.107	3.116	1.49321	0.0004	0.0439441
XLOC_013616	DNAH2	19:28025442-28134625	0.338	0.095	-1.82699	0.0004	0.0439441
XLOC_034604	ENSBTAG00000001219	8:73085153-73094602	100.505	54.387	-0.885947	0.0004	0.0439441
XLOC_026414	ENSBTAG00000032057	3:28623390-28623702	85.946	28.080	-1.61392	0.0004	0.0439441
XLOC_004506	GAREML	11:73290650-73307412	8.342	4.129	-1.01437	0.0004	0.0439441
XLOC_028262	TRPV6	4:106996848-107019730	62.942	29.255	-1.10533	0.0004	0.0439441
XLOC_030454	CFI	6:16764539-16815352	36.794	68.717	0.901191	0.00045	0.0481429
XLOC_030738	CXCL6	6:90645963-90648076	1.297	3.510	1.43609	0.00045	0.0481429
XLOC_014243	ENSBTAG00000038770	19:6313770-6338246	0.506	2.240	2.14498	0.00045	0.0481429
XLOC_027324	HOX3A	4:69357066-69389906	5.556	1.613	-1.78461	0.00045	0.0481429

XLOC_027324	HOX4A	4:69357066-69389906	5.556	1.613	-1.78461	0.00045	0.0481429
XLOC_027711	Unknown	4:119468337-119469175	15.402	6.549	-1.23368	0.00045	0.0481429
XLOC_022491	ENSBTAG00000023955	26:16231609-16290416	180.133	83.961	-1.10128	0.0005	0.051085
XLOC_008888	ENSBTAG00000040339	15:50265099-50270713	1.597	0.272	-2.55445	0.0005	0.051085
XLOC_012103	FLT3LG	18:56383528-56393411	19.401	9.345	-1.05391	0.0005	0.051085
XLOC_016439	FN1	2:103881401-103950584	1.888	4.767	1.33606	0.0005	0.051085
XLOC_025585	LOC507055	3:54956543-54958432	61.256	33.653	-0.864118	0.0005	0.051085
XLOC_022896	NT5C2	26:23983196-24080600	8.488	4.001	-1.08503	0.0005	0.051085
XLOC_028584	RND1	5:31090613-31097555	6.034	2.544	-1.24584	0.0005	0.051085
XLOC_006498	THBD	13:42217370-42221004	0.206	0.742	1.85076	0.0005	0.051085
XLOC_028051	Unknown	4:74990606-74995166	0.103	0.510	2.31133	0.0005	0.051085
XLOC_030456	CASP6	6:16847110-16867961	70.999	126.985	0.838788	0.00055	0.0553631
XLOC_004992	SLC7A1	12:30992433-31024002	2.946	5.718	0.956567	0.00055	0.0553631
XLOC_001929	THBS1	10:35314024-35329297	0.429	1.402	1.70773	0.00055	0.0553631
XLOC_004349	LOC100300483	11:49060220-49140167	186.848	433.100	1.21283	0.0006	0.0595165
XLOC_007960	Unknown	15:29533798-29538126	0.025	0.232	3.23367	0.0006	0.0595165
XLOC_018948	UROC1	22:61133593-61161401	20.580	9.627	-1.09601	0.0006	0.0595165
XLOC_022866	PKD2L1	26:21055303-21132239	1.232	0.291	-2.0826	0.00065	0.0632481
XLOC_029038	STYK1	5:99373716-99442367	112.307	51.437	-1.12656	0.00065	0.0632481
XLOC_034455	TRPM6	8:51116465-51286550	1.328	0.511	-1.37728	0.00065	0.0632481
XLOC_025578	Unknown	3:54896671-54897535	21.419	9.799	-1.12814	0.00065	0.0632481
XLOC_020435	ENSBTAG00000035959	23:29961278-29962663	3.009	8.440	1.48792	0.0007	0.0665293
XLOC_020574	MYLIP	23:40770314-40791061	51.587	28.781	-0.841921	0.0007	0.0665293
XLOC_014748	PDK2	19:37177680-37193942	39.490	22.124	-0.835898	0.0007	0.0665293
XLOC_003655	Unknown	11:69137679-69141496	8.991	4.891	-0.878258	0.0007	0.0665293
XLOC_017568	Unknown	21:27054891-27108980	17.279	9.185	-0.91166	0.0007	0.0665293
XLOC_016551	ECEL1	2:120925688-120933290	8.650	2.438	-1.8273	0.00075	0.0690338
XLOC_009998	F8	16:37964915-38046205	2.631	5.022	0.932672	0.00075	0.0690338
XLOC_017655	GZMB	21:35135770-35137852	81.466	161.009	0.982878	0.00075	0.0690338
XLOC_001846	RNASE1	10:26388179-26389761	1.885	4.844	1.36184	0.00075	0.0690338
XLOC_029290	TEF	5:113028011-113041319	8.414	4.382	-0.941086	0.00075	0.0690338
XLOC_021026	Unknown	24:7206795-7216215	0.601	0.083	-2.85328	0.00075	0.0690338
XLOC_004382	Unknown	11:54460938-54461063	1609.640	503.702	-1.67609	0.00075	0.0690338

XLOC_016066	COL3A1	2:7317293-7357126	7.010	15.733	1.16635	0.0008	0.0720141
XLOC_018461	CRIP1	21:71390599-71392110	3769.250	2059.510	-0.871979	0.0008	0.0720141
XLOC_005143	ENSBTAG00000047360	12:74068056-74250762	10.862	21.113	0.958839	0.0008	0.0720141
XLOC_024062	Unknown	29:5326644-5328813	3.043	1.225	-1.31242	0.0008	0.0720141
XLOC_028668	Unknown	5:44453408-44453650	2.818	11.383	2.01425	0.0008	0.0720141
XLOC_007210	PKIA	14:43880454-43980604	110.009	58.227	-0.917868	0.00085	0.0761794
XLOC_008326	CAPN5	15:57256155-57313834	48.600	28.437	-0.773207	0.0009	0.0775975
XLOC_019686	CLIC1	23:27375622-27398934	300.451	170.648	-0.816102	0.0009	0.0775975
XLOC_029037	CSDA	5:99335354-99360895	7.186	14.636	1.02617	0.0009	0.0775975
XLOC_024377	CTSW	29:44661464-44665100	9.364	3.940	-1.2488	0.0009	0.0775975
XLOC_025560	ENSBTAG00000014857	3:54031911-54060885	19.154	9.352	-1.03429	0.0009	0.0775975
XLOC_011205	ERP29	17:64419026-64426503	30.672	53.511	0.802912	0.0009	0.0775975
XLOC_013075	NKG7	18:57812520-57815305	59.631	32.325	-0.8834	0.0009	0.0775975
XLOC_036031	SLC6A8	X:39828307-39836767	110.168	55.858	-0.979876	0.0009	0.0775975
XLOC_024429	Unknown	29:46054614-46057355	25.412	11.894	-1.09526	0.0009	0.0775975
XLOC_031842	ANGPTL4	7:18236516-18243581	62.363	35.949	-0.794732	0.00095	0.0808846
XLOC_032797	bta-mir-24-2	7:12980796-12981713	4.193	1.658	-1.33838	0.00095	0.0808846
XLOC_028541	SLC11A2	5:28885334-29122001	64.859	153.142	1.23948	0.00095	0.0808846
XLOC_010986	CCRN4L	17:18892222-18910926	4.162	1.793	-1.21484	0.001	0.0827287
XLOC_015993	ENSBTAG00000013039	2:133318093-133323729	8.847	21.225	1.26254	0.001	0.0827287
XLOC_030192	FBXL14	5:108602403-108613565	7.796	4.056	-0.942582	0.001	0.0827287
XLOC_028007	PRR15	4:67039127-67042539	72.435	40.417	-0.84171	0.001	0.0827287
XLOC_028621	SLC38A2	5:34028552-34042996	18.843	32.720	0.796119	0.001	0.0827287
XLOC_022955	Unknown	26:35438198-35439355	1.769	0.431	-2.03675	0.001	0.0827287
XLOC_019094	Unknown	22:29147740-29196185	1.267	0.513	-1.30428	0.001	0.0827287
XLOC_020215	LRRC73	23:17025825-17028692	33.296	15.570	-1.09662	0.00105	0.0848051
XLOC_001492	TFF2	1:144176744-144180011	180.841	347.087	0.940574	0.00105	0.0848051
XLOC_014479	TLCD2	19:23373520-23378706	1.406	0.493	-1.51184	0.00105	0.0848051
XLOC_027689	Unknown	4:117869785-117873545	0.345	0.070	-2.31148	0.00105	0.0848051
XLOC_000114	Unknown	1:36700313-36738891	1.392	0.107	-3.69833	0.00105	0.0848051
XLOC_010285	Unknown	16:68995795-68995955	45.207	7.524	-2.58692	0.00105	0.0848051
XLOC_016743	ENSBTAG00000005140	2:133631935-133644698	13.401	6.142	-1.12564	0.0011	0.0884937
XLOC_013402	CCL3	19:14673889-14675910	6.812	2.683	-1.34424	0.00115	0.0907301

XLOC_026899	LOC101907688	3:104408540-104421736	42.007	14.071	-1.57786	0.00115	0.0907301
XLOC_022922	PRAP1	26:25881751-25885322	5374.250	2567.940	-1.06545	0.00115	0.0907301
XLOC_028649	PTPRR	5:42837596-43112802	9.160	4.645	-0.979637	0.00115	0.0907301
XLOC_031389	SPARCL1	6:104149823-104203225	3.956	9.811	1.31036	0.00115	0.0907301
XLOC_007846	MMP1	15:6123995-6141339	3.000	7.545	1.33073	0.0012	0.0935908
XLOC_003623	SLC1A4	11:63290421-63395507	2.070	4.408	1.09023	0.0012	0.0935908
XLOC_007475	Unknown	14:3837013-3837101	6623.610	19467.000	1.55534	0.0012	0.0935908
XLOC_023123	Unknown	27:14323896-14333588	0.766	0.299	-1.35722	0.00125	0.0963868
XLOC_003966	Unknown	11:105094032-10511459	1.101	0.305	-1.85179	0.00125	0.0963868
XLOC_027941	Unknown	4:50369390-50370853	3.231	1.254	-1.3654	0.00125	0.0963868
XLOC_008731	CT	15:38041579-38044615	1.085	0.081	-3.74954	0.0013	0.0980229
XLOC_013562	CXCL16	19:27243299-27253097	402.248	231.093	-0.79961	0.0013	0.0980229
XLOC_026164	FCER1A	3:10539987-10546232	4.642	10.016	1.10953	0.0013	0.0980229
XLOC_004066	NEURL3	11:2486725-2494849	0.615	0.199	-1.62849	0.0013	0.0980229
XLOC_037305	TMSB4	X:140973701-140975767	1991.510	1126.720	-0.821731	0.0013	0.0980229
XLOC_011409	Unknown	18:2904735-2907471	0.174	0.587	1.75272	0.0013	0.0980229

Supplementary Table S3: List of significantly enriched Ingenuity Canonical Pathways with genes differentially expressed between jejunal mucosa of calves with a restricted and *ad libitum* milk replacer diet				
**Ingenuity Canonical Pathways**	**-log(p-value)**	**Ratio**	**z-score**	**Molecules**
Agranulocyte Adhesion and Diapedesis	1.09E+01	8.47E-02		SELL,CXCL11,FN1,CLDN15,CCL5,MMP25,CCL3,MYH7B,CXCL6,CXCL10,CXCL16,CCL4,CLDN4,XCL1,ACTA1,MMP1
Granulocyte Adhesion and Diapedesis	9.17E+00	7.91E-02		SELL,CXCL11,CLDN15,IL1RL1,CCL5,MMP25,CCL3,CXCL6,CXCL10,CXCL16,CCL4,CLDN4,XCL1,MMP1
Pathogenesis of Multiple Sclerosis	8.19E+00	5.56E-01		CXCL10,CXCL11,CCL4,CCL5,CCL3
IL-17A Signaling in Gastric Cells	7.15E+00	2.40E-01		CXCL10,FOS,CXCL11,JUN,CCL5,MAPK11
Complement System	4.74E+00	1.35E-01		SERPING1,C4BPA,CFI,C6,C8G
CCR5 Signaling in Macrophages	4.46E+00	8.70E-02		FOS,JUN,CCL4,CCL5,CCL3,MAPK11
Bupropion Degradation	4.18E+00	1.60E-01		CYP1A1,CYP4B1,CYP2C18,CYP2C9
VDR/RXR Activation	4.16E+00	7.69E-02	-1.348	CXCL10,TRPV6,IL1RL1,CCL5,VDR,THBD
Acetone Degradation I (to Methylglyoxal)	4.04E+00	1.48E-01		CYP1A1,CYP4B1,CYP2C18,CYP2C9
IL-17A Signaling in Fibroblasts	3.59E+00	1.14E-01		FOS,JUN,MAPK11,MMP1
Estrogen Biosynthesis	3.41E+00	1.03E-01		CYP1A1,CYP4B1,CYP2C18,CYP2C9
Chemokine Signaling	3.37E+00	7.04E-02	-2.236	FOS,JUN,CCL4,CCL5,MAPK11
Role of MAPK Signaling in the Pathogenesis of Influenza	3.34E+00	6.94E-02		CXCL10,PLB1,CCL5,MAPK11,PLA2G2A
Differential Regulation of Cytokine Production in Macrophages and T Helper Cells by I	3.29E+00	1.67E-01		CCL4,CCL5,CCL3
Toll-like Receptor Signaling	3.29E+00	6.76E-02		UBD,FOS,JUN,IL1RL1,MAPK11
Role of Hypercytokinemia/hyperchemokinemia in the Pathogenesis of Influenza	3.24E+00	9.30E-02		CXCL10,CCL4,CCL5,CCL3
Tight Junction Signaling	3.12E+00	4.19E-02		FOS,JUN,CLDN15,CLDN4,YBX3,MYH7B,ACTA1
Acute Phase Response Signaling	3.09E+00	4.14E-02	-0.447	FOS,SERPING1,FN1,JUN,F8,C4BPA,MAPK11
Differential Regulation of Cytokine Production in Intestinal Epithelial Cells by IL-17A a	2.97E+00	1.30E-01		CCL4,CCL5,CCL3
Sertoli Cell-Sertoli Cell Junction Signaling	2.96E+00	3.93E-02		JUN,CLDN15,CLDN4,YBX3,TUBB4A,MAPK11,ACTA1
Nicotine Degradation III	2.87E+00	7.41E-02		CYP1A1,CYP4B1,CYP2C18,CYP2C9
Endothelin-1 Signaling	2.84E+00	3.74E-02	-1.134	CASP6,FOS,JUN,PLB1,ADCY6,MAPK11,PLA2G2A
ILK Signaling	2.72E+00	3.57E-02	-1.89	FOS,FN1,JUN,RHOD,TMSB10/TMSB4X,MYH7B,ACTA1
Tryptophan Degradation to 2-amino-3-carboxymuconate Semialdehyde	2.67E+00	2.50E-01		IDO1,IDO2
Intrinsic Prothrombin Activation Pathway	2.67E+00	1.03E-01		F8,THBD,COL3A1
Melatonin Degradation I	2.65E+00	6.45E-02		CYP1A1,CYP4B1,CYP2C18,CYP2C9
Nicotine Degradation II	2.62E+00	6.35E-02		CYP1A1,CYP4B1,CYP2C18,CYP2C9
Role of IL-17A in Arthritis	2.52E+00	5.97E-02		CCL5,MAPK11,CXCL6,MMP1
Superpathway of Melatonin Degradation	2.52E+00	5.97E-02		CYP1A1,CYP4B1,CYP2C18,CYP2C9
IL-10 Signaling	2.50E+00	5.88E-02		FOS,JUN,IL1RL1,MAPK11
Inhibition of Angiogenesis by TSP1	2.47E+00	8.82E-02		JUN,THBS1,MAPK11
Coagulation System	2.43E+00	8.57E-02		F8,SERPINA5,THBD
Glucocorticoid Receptor Signaling	2.37E+00	2.79E-02		FOS,JUN,SGK1,PCK1,CCL5,CCL3,MAPK11,MMP1
Renin-Angiotensin Signaling	2.36E+00	4.17E-02		FOS,JUN,ADCY6,CCL5,MAPK11
April Mediated Signaling	2.33E+00	7.89E-02		FOS,JUN,MAPK11
B Cell Activating Factor Signaling	2.27E+00	7.50E-02		FOS,JUN,MAPK11
MIF Regulation of Innate Immunity	2.24E+00	7.32E-02		FOS,JUN,PLA2G2A
GNRH Signaling	2.22E+00	3.88E-02	-1.342	FOS,JUN,EGR1,ADCY6,MAPK11
Hepatic Fibrosis / Hepatic Stellate Cell Activation	2.22E+00	3.28E-02		FN1,IL1RL1,CCL5,MYH7B,MMP1,COL3A1
UVC-Induced MAPK Signaling	2.21E+00	7.14E-02		FOS,JUN,MAPK11
IL-17 Signaling	2.15E+00	4.71E-02		CXCL10,CXCL11,JUN,MAPK11
Role of IL-17F in Allergic Inflammatory Airway Diseases	2.15E+00	6.82E-02		CXCL10,CCL4,CXCL6
iNOS Signaling	2.15E+00	6.82E-02		FOS,JUN,MAPK11
RAR Activation	2.14E+00	3.16E-02		FOS,JUN,ALDH1A3,ADCY6,MAPK11,MMP1
TGF-Î² Signaling	2.12E+00	4.60E-02		FOS,JUN,VDR,MAPK11
NAD biosynthesis II (from tryptophan)	2.12E+00	1.33E-01		IDO1,IDO2
NRF2-mediated Oxidative Stress Response	2.11E+00	3.11E-02		FOS,JUN,ERP29,JUNB,ACTA1,GSTO1
Communication between Innate and Adaptive Immune Cells	2.08E+00	4.49E-02		CXCL10,CCL4,CCL5,CCL3

Aryl Hydrocarbon Receptor Signaling	2.08E+00	3.57E-02		FOS,CYP1A1,JUN,ALDH1A3,GSTO1
IL-1 Signaling	2.05E+00	4.40E-02	-1	FOS,JUN,ADCY6,MAPK11
TNFR1 Signaling	2.02E+00	6.12E-02		CASP6,FOS,JUN
Leukocyte Extravasation Signaling	1.94E+00	2.86E-02	-1	CLDN15,CLDN4,MMP25,MAPK11,ACTA1,MMP1
ErbB Signaling	1.94E+00	4.08E-02		FOS,JUN,MAPK11,EREG
CDK5 Signaling	1.93E+00	4.04E-02	-1	LAMC1,EGR1,ADCY6,MAPK11
Antioxidant Action of Vitamin C	1.87E+00	3.88E-02		PLB1,MAPK11,PLA2G2A,GSTO1
LPS/IL-1 Mediated Inhibition of RXR Function	1.84E+00	2.71E-02		GAL3ST2,JUN,ALDH1A3,IL1RL1,CYP2C9,GSTO1
Tryptophan Degradation III (Eukaryotic)	1.83E+00	9.52E-02		IDO1,IDO2
CXCR4 Signaling	1.79E+00	3.03E-02	-2	FOS,JUN,RHOD,EGR1,ADCY6
Corticotropin Releasing Hormone Signaling	1.76E+00	3.60E-02		FOS,JUN,ADCY6,MAPK11
IL-22 Signaling	1.72E+00	8.33E-02		IL22RA1,MAPK11
Tumoricidal Function of Hepatic Natural Killer Cells	1.72E+00	8.33E-02		CASP6,GZMB
HIF1Î± Signaling	1.71E+00	3.48E-02		JUN,MMP25,MAPK11,MMP1
UVB-Induced MAPK Signaling	1.68E+00	4.55E-02		FOS,JUN,MAPK11
PPARÎ±/RXRÎ± Activation	1.66E+00	2.81E-02	-1	JUN,IL1RL1,ADCY6,CYP2C18,CYP2C9
Calcium Signaling	1.66E+00	2.81E-02		RCAN1,TRPV6,MYH7B,ACTA1,GRINA
EGF Signaling	1.64E+00	4.41E-02		FOS,JUN,MAPK11
Colorectal Cancer Metastasis Signaling	1.63E+00	2.43E-02	-0.816	FOS,JUN,RHOD,ADCY6,MMP25,MMP1
Agrin Interactions at Neuromuscular Junction	1.63E+00	4.35E-02		LAMC1,JUN,ACTA1
L-serine Degradation	1.58E+00	3.33E-01		SDS
Atherosclerosis Signaling	1.57E+00	3.15E-02		PLB1,PLA2G2A,MMP1,COL3A1
IL-6 Signaling	1.57E+00	3.15E-02	-1	FOS,JUN,IL1RL1,MAPK11
TNFR2 Signaling	1.56E+00	6.90E-02		FOS,JUN
4-1BB Signaling in T Lymphocytes	1.51E+00	6.45E-02		JUN,MAPK11
HMGB1 Signaling	1.51E+00	3.01E-02	-2	FOS,JUN,RHOD,MAPK11
CD40 Signaling	1.49E+00	3.85E-02		FOS,JUN,MAPK11
Cytotoxic T Lymphocyte-mediated Apoptosis of Target Cells	1.48E+00	6.25E-02		CASP6,GZMB
Arsenate Detoxification I (Glutaredoxin)	1.45E+00	2.50E-01		GSTO1
Ascorbate Recycling (Cytosolic)	1.45E+00	2.50E-01		GSTO1
Renal Cell Carcinoma Signaling	1.45E+00	3.70E-02		UBD,FOS,JUN
Interferon Signaling	1.39E+00	5.56E-02		IFI6,ISG15
LPS-stimulated MAPK Signaling	1.38E+00	3.49E-02		FOS,JUN,MAPK11
Bladder Cancer Signaling	1.37E+00	3.45E-02		THBS1,MMP25,MMP1
Xenobiotic Metabolism Signaling	1.37E+00	2.10E-02		GAL3ST2,CYP1A1,ALDH1A3,CYP2C9,MAPK11,GSTO1
Tetrapyrrole Biosynthesis II	1.36E+00	2.00E-01		ALAD
Inhibition of Matrix Metalloproteases	1.33E+00	5.13E-02		MMP25,MMP1
eNOS Signaling	1.30E+00	2.58E-02		AQP7,ADCY6,SLC7A1,AQP1
PPAR Signaling	1.30E+00	3.23E-02		FOS,JUN,IL1RL1

Supplementary Table S5: List of differentially expressed genes classified as enzyme, peptidase or transporter as indicated by the Ingenuity Knowledge Database

Symbol	Entrez Gene Name	Type(s)
ISG20	interferon stimulated exonuclease gene 20kDa	enzyme
FBXL14	F-box and leucine-rich repeat protein 14	enzyme
ABHD17C	abhydrolase domain containing 17C	enzyme
ALAD	aminolevulinate dehydratase	enzyme
MYLIP	myosin regulatory light chain interacting protein	enzyme
B4GALNT2	beta-1,4-N-acetyl-galactosaminyltransferase 2	enzyme
CYP4B1	cytochrome P450 family 4 subfamily B member 1	enzyme
GSTO1	glutathione S-transferase omega 1	enzyme
GAL3ST2	galactose-3-O-sulfotransferase 2	enzyme
IDO2	indoleamine 2,3-dioxygenase 2	enzyme
CA9	carbonic anhydrase 9	enzyme
CPO	carboxypeptidase O	enzyme
REM1	RAS (RAD and GEM)-like GTP-binding 1	enzyme
PNLIPRP2	pancreatic lipase related protein 2 (gene/pseudogene)	enzyme
ADCY6	adenylate cyclase 6	enzyme
TGM3	transglutaminase 3	enzyme
PLA2G2A	phospholipase A2 group IIA	enzyme
PLB1	phospholipase B1	enzyme
CYP2C18	cytochrome P450 family 2 subfamily C member 18	enzyme
ALDH1A3	aldehyde dehydrogenase 1 family member A3	enzyme
SDS	serine dehydratase	enzyme
CYP1A1	cytochrome P450 family 1 subfamily A member 1	enzyme
DDAH1	dimethylarginine dimethylaminohydrolase 1	enzyme
DDAH2	dimethylarginine dimethylaminohydrolase 2	enzyme
GBP2	guanylate binding protein 2	enzyme
RHOD	ras homolog family member D	enzyme
RNASE1	ribonuclease A family member 1, pancreatic	enzyme
FN1	fibronectin 1	enzyme
PLA2G2A	phospholipase A2 group IIA	enzyme
ENPP2	ectonucleotide pyrophosphatase/phosphodiesterase 2	enzyme

GBP6	guanylate binding protein family member 6	enzyme
UROC1	urocanate hydratase 1	enzyme
CYP2C9	cytochrome P450 family 2 subfamily C member 9	enzyme
IDO1	indoleamine 2,3-dioxygenase 1	enzyme
RGN	regucalcin	enzyme
RND1	Rho family GTPase 1	enzyme
AGMO	alkylglycerol monooxygenase	enzyme
NEURL3	neuralized E3 ubiquitin protein ligase 3	enzyme
GZMB	granzyme B	peptidase
F8	coagulation factor VIII	peptidase
CFI	complement factor I	peptidase
CASP6	caspase 6	peptidase
CAPN5	calpain 5	peptidase
CPA1	carboxypeptidase A1	peptidase
MMP25	matrix metallopeptidase 25	peptidase
MMP1	matrix metallopeptidase 1	peptidase
MME	membrane metallo-endopeptidase	peptidase
CTSW	cathepsin W	peptidase
ECEL1	endothelin converting enzyme-like 1	peptidase
SLC6A8	solute carrier family 6 member 8	transporter
SLC7A1	solute carrier family 7 member 1	transporter
NPC1L1	NPC1 like 1	transporter
SLC28A1	solute carrier family 28 member 1	transporter
ERP29	endoplasmic reticulum protein 29	transporter
SLC38A2	solute carrier family 38 member 2	transporter
AQP7	aquaporin 7	transporter
C8G	complement component 8, gamma polypeptide	transporter
SLC45A3	solute carrier family 45 member 3	transporter
TFRC	transferrin receptor	transporter
SLC6A14	solute carrier family 6 member 14	transporter
STEAP1	six transmembrane epithelial antigen of the prostate 1	transporter
SLC11A2	solute carrier family 11 member 2	transporter
AQP1	aquaporin 1 (Colton blood group)	transporter

SLC1A4	solute carrier family 1 member 4	transporter
MFSD2A	major facilitator superfamily domain containing 2A	transporter
FLVCR2	feline leukemia virus subgroup C cellular receptor family memb	transporter
SLCO2A1	solute carrier organic anion transporter family member 2A1	transporter

Supplementary Table S6. Nutrient and chemical composition of the milk replacer (MR) and concentrate (CON)[1]

Item	Milk replacer[2]	Concentrates[3]
Dry Matter (DM) [%]	96.8	86.6
Crude Protein [% of DM]	21.7	21.0
Crude Fat [% of DM]	18.6	4.2
Crude Fiber [% of DM]	0.2	5.9
ADFOM[4] [% of DM]	n.d.[7]	8.1
aNDFOM[4] [% of DM]	n.d.	16.5
Ash [% of DM]	7.3	7.0
NFE[5]	52.1	61.9
Total sugar [% of DM]	44.9	n.d.
ME MJ/kg DM[6]	18.34	13.15
Ca [% of DM]	1.17	1.22
P [% of DM]	0.65	0.66
Na [% of DM]	0.47	0.29
K [% of DM]	1.43	1.21
Lysine [% of DM]	1.8	n.d.
Methionine [% of DM]	0.48	n.d.

[1]Date were first presented by [28].

[2]Ingredients of MR: 50 % skim milk powder, 25 % whey powder, 16.5 % vegetable oil, 3 % wheat powder.

[3]Ingredients of CON: 35.0 % corn, 23.0 % soybean meal, 13.0 % beet pulp dried with molasses, 7.5 % wheat, 6.0 % barley, 3.2 % rapeseed meal, 3.0 % linseed meal, 3.0 % beet pulp, 2.0 % wheat bran, 0.09 % calcium carbonate, 0.05 % mono-calcium phosphate, 0.0075 % ferrous sulphate.

[4]Acid detergent fiber (ADF) and neutral detergent fiber (NDF) are expressed exclusive of residual ash. NDF was assayed with a heat stable amylase [28].

[5]Nitrogen-free extract (NFE), calculated as NFE = 100 - (crude protein + crude fat + crude fiber + ash).

[6]ME, calculated using the equation: ME MJ/kg DM = (24.2*crude protein + 36.6*crude fat + 17.0*total sugar)/100*0.97*0.96.

[7]n.d. = not determined
